# ﻿*Adelphomyia* crane flies (Diptera, Limoniidae) of Korea with identification key for all Palaearctic species

**DOI:** 10.3897/zookeys.1217.115627

**Published:** 2024-10-30

**Authors:** Sigitas Podenas, Sun-Jae Park, Changhwan Bae

**Affiliations:** 1 Nature Research Centre, Akademijos str. 2, LT-08412 Vilnius, Lithuania Nature Research Centre Vilnius Lithuania; 2 Life Sciences Centre of Vilnius University, Sauletekio str. 7, LT-10257 Vilnius, Lithuania Life Sciences Centre of Vilnius University Vilnius Lithuania; 3 Climate Change and Environmental Biology Research Division, National Institute of Biological Resources, Incheon 404-708, Republic of Korea National Institute of Biological Resources Incheon Republic of Korea; 4 Species Diversity Research Division, National Institute of Biological Resources, Incheon 404-708, Republic of Korea Species Diversity Research Division, National Institute of Biological Resources Incheon Republic of Korea

**Keywords:** East Palaearctic, habitat, key, Limnophilinae, taxonomy

## Abstract

Limnophilinae crane flies belonging to the genus *Adelphomyia* Bergroth, 1891 of the Korean Peninsula were studied starting from 1937, but only one species *A.macrotrichiata* (Alexander, 1923) has been recorded from North Korea so far. The genus was unknown from South Korea. Four species were found during our studies on the Peninsula, one of them from Jeju Island described as new, *Adelphomyiajejuana* Podenas, **sp. nov.** Three species are recorded from both northern and southern parts of the Peninsula. Specimens on which was based the record of *A.macrotrichiata* from North Korea was misidentified and no more specimens were collected; therefore, *A.macrotrichiata* is deleted from the Korean species list. Habitat, elevation range, and seasonality data is presented for each species. Images of taxonomically important morphological characters, and an identification key for all Palaearctic species of the genus *Adelphomyia* are presented. Distribution maps are presented for all Korean species.

## ﻿Introduction

Crane flies belonging to the genus *Adelphomyia* Bergroth, 1891 are easily recognised by the densely trichiated distal wing cells and nearly translucent wings with greatly reduced dark pattern except the distinct stigma. Adults fly in shaded places close to streams (Salmela and Härmä 2004), but larvae and other preimaginal stages are still unknown for the genus. Adults usually are collected together with other Limoniidae crane flies that prevail in wet areas under tree canopies. Despite many *Adelphomyia* specimens were collected in 1937–1939, only one species, *A.macrotrichiata* (Alexander, 1923) was recorded from the north of the Korean Peninsula and no species were known from South Korea. Our studies of the museum specimens and of specimens collected by ourselves in 2012–2019 revealed four species of *Adelphomyia*. Three of them are widely distributed in North and South Koreas, and one species from Jeju Island is new. Despite many specimens collected during field trips, *A.macrotrichiata* was not found in South Korea, it was not found also in additional material available from the museum collections.

## ﻿Materials and methods

Despite the many museum collections that were examined, *Adelphomyia* crane flies from the Korean Peninsula were found only at the National Institute of Biological Resources (**NIBR**), Incheon, South Korea; The Snow Entomological Museum, University of Kansas, Lawrence, KS, USA (**SMEK**); and at the National Museum of Natural History, Smithsonian Institution, Washington, DC, USA (**USNM**). Comparative material from Lithuania for *A.punctum* (Meigen, 1818) was used from the collections of the Nature Research Centre (**NRC**), Vilnius, Lithuania.

Adults were collected in various ways, including by insect nets, with Malaise traps, LED light traps, black light traps, Mosquito Magnet® traps (Pro Model, Woodstream Corp., Lititz, PA), New Jersey (NJ) traps, and at light sources. The collected specimens were dry mounted laterally on paper points. Wet specimens were preserved in 96% ethanol (EtOH). Some male wings were slide-mounted in Euparal and photographed. Dissected male genitalia were cleared in 10% KOH and preserved in microvials with glycerol.

Information on the examined material is given according to the journal requirements, thus altitudes are given in metric system regardless of the system applied for the label. For specimens collected by SP and his colleagues, the date on the label is followed by a number in brackets, the number referring to locality: different localities where insects were collected on the same date were given separate numbers and all information from those localities, whether in the field notes, database, photographs, and other locality information, were marked with this specific number. Specimens are arranged according to the collecting date.

Specimens were examined with an Olympus SZX10 dissecting microscope and Nikon Eclipse Ti microscope. Photographs were taken with a Canon R5 camera through a Canon MP-E 65 mm macro lens and through Mitutoyo M Plan apo 10× lens mounted on the same camera at Nature Research Centre, Vilnius, Lithuania.

The terminology of adult morphological features generally follows that of Cumming and Wood (2017), while terminology of wing venation follows de Jong (2017).

## ﻿Taxonomy

### ﻿ Adelphomyia

Taxon classificationAnimaliaDipteraLimoniidae

Bergroth, 1891

9756E0BC-10F6-590E-8240-4B0B21DEAFF2

Adelphomyia Bergroth, 1891: 134; Savchenko and Krivolutskaya 1976: 57; Savchenko 1983: 49; Savchenko 1986: 273–275; Savchenko 1989: 76–79.
Limnophila (Tricholimnophila) Alexander, 1928: 476–477.
Limnophila (Adelphomyia) : Alexander 1938: 324; Ishida 1959: 2.

#### Type species.

*Adelphomyiahelvetica* Bergroth, 1891 (= *punctum* Meigen, 1818) (West and East Palaearctic).

#### Type locality.

Weissenburg, Canton Bern, Switzerland.

#### Description.

Medium-sized crane flies with body length 3.9–8.4 mm and wing length 5.5–8.8 mm. Colouration varies from pale yellow to dark brown or black (Figs 25, 40, 44).

***Head.*** Rounded posteriorly. Antenna with 14-segmented flagellum. Flagellomeres slightly elongate or oval, covered with short pubescence, verticils variable, up to 2.5× as long as respective segment.

**Figures 1–3.**
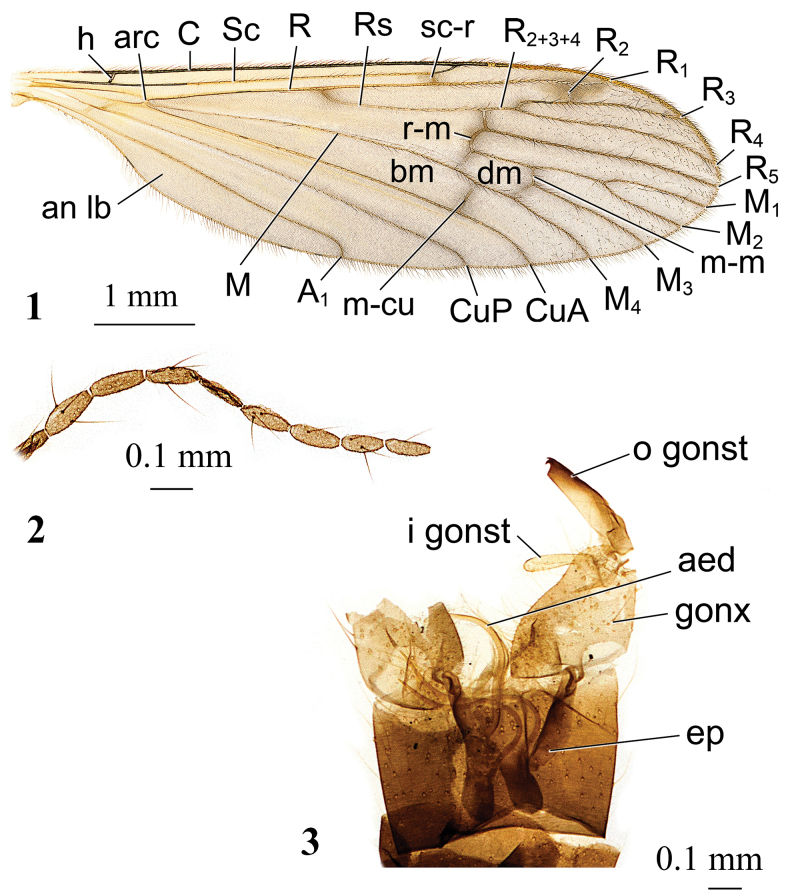
*Adelphomyiaacicularisacicularis* (Alexander, 1954), male **1** wing, paratype **2** fragment of antennal flagellum, paratype **3** genitalia, dorsal view, holotype. Abbreviations: *A_1_* – first branch of anal vein; aed – aedeagus; an lb – anal lobe; *arc* – arculus; *bm* – basal medial cell; *C* – costal vein; *CuA* – anterior branch of cubital vein; *CuP* – posterior branch of cubital vein; *dm* – discal medial cell; ep – epandrium (ninth tergite); gonx – gonocoxite; *h* – humeral vein; i gonst – inner gonostylus; *M* – medial vein, or media; *M_1_* – first branch of media; *M_2_* – second branch of media; *M_3_* – third branch of media; *M_4_* – fourth branch of media; *m-cu* – medial-cubital cross-vein; *m-m* – medial cross-vein; o gonst – outer gonostylus; *R* – radius, or radial vein; *R_1_* – anterior branch of radius; *R_2_* – second branch of radius; *R_2+3+4_* – stem of radial branches *R_2_*, *R_3_* and *R_4_*; *R_3_* – lower branch of second branch of radius; *R_4_* – upper branch of third branch of radius; *R_5_* – lower branch of third branch of radius; *r-m* – radio-medial cross-vein; *Rs* – radial sector; *Sc* – subcostal vein; *sc-r* – subcostal-radial cross-vein.

***Thorax.*** Frontal margin of pronotum straight. Mesonotal prescutum with distinct tubercular pits and pseudosutural fovea. Katepisternum bare, without setae. Meron small. Middle and posterior coxae close to each other. Wing (Figs 1, 5, 9, 12, 15, 17, 22, 24, 27, 29, 34, 36, 38, 41, 45) comparatively wide, no pattern or with darkening surrounding only cross-veins except stigma. *Arculus* present, vein *Sc* reaching wing margin slightly before branching point of *Rs*, *sc-r* approximately its own length before tip of *Sc. R_1_* elongate, *R_2_* 2–3× its own length before tip of *R_1_*. Radial sector long, cell *r_3_* long with short stem. Cell *m_1_* usually long, but sometimes missing completely (e.g., one wing of *A.satsumicola* (Alexander, 1930) holotype). Discal cell always present, usually elongate. Cross-vein *m-cu* far beyond branching point of *M.* Anal vein reaching wing margin at approximately same level as base of *Rs.* Anal angle wide. Distal wing cells always with macrotrichiae. Frontal tibia with single spur, tibiae of second and third pairs of legs with two spurs each.

***Abdomen.*** Tergites with two transverse indentations frontally. Male terminalia approximately as wide as rest abdominal segments. Epandrium (ninth tergum) with two small lobes at the middle of posterior margin. Gonocoxite simple: elongate with no additional lobes, two pairs of elongate, narrow gonostyles. According to Savchenko (1986) interbases are missing, still the structure is present (Figs 7, 20, 32, 43), in addition, Ribeiro (2008) showed the structure is clearly noticeable in Limnophilinae. Aedeagus long, narrow, one pair of elongate parameres, but length varies widely depending on species. Ovipositor (Figs 8, 21, 33, 46) with long, narrow cercus and hypogynial valve.

Twenty-five species of *Adelphomyia* are known worldwide (Oosterbroek 2024), 13 of them occur in Oriental Region and 12 (one of them with two subspecies) in East Palaearctic. Only *A.punctum* (Meigen, 1818) has a wide distribution, occurring in East and West Palaearctic.

##### ﻿List of Palaearctic *Adelphomyia* species (species from Korean Peninsula marked with asterisk)

*Adelphomyiaacicularisacicularis* (Alexander, 1954) (Figs 1–3)

**Figures 4–8.**
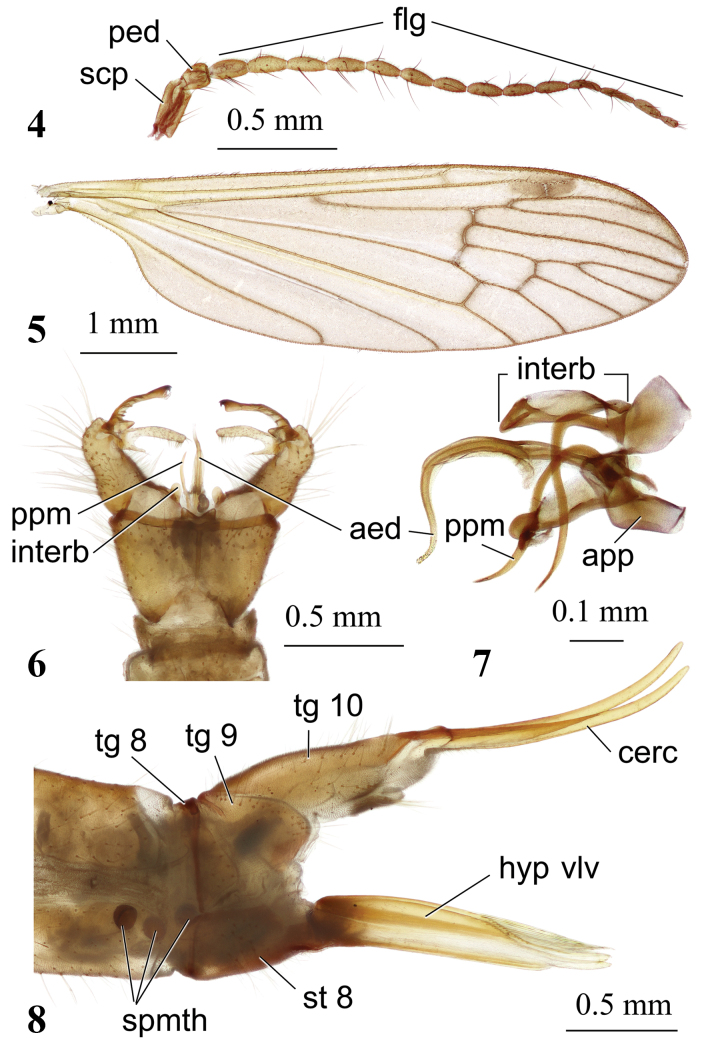
*Adelphomyiaacicularisbidens* Savchenko, 1983 **4** male antenna **5** male wing **6** male genitalia, dorsal view **7** aedeagal complex, lateral view **8** ovipositor, lateral view. Abbreviations: aed – aedeagus; app – anterior part of paramere; cerc – cercus; flg – flagellum; hyp vlv – hypogynial valve ; interb – interbase; ped – pedicel; ppm – posterior part of paramere; scp – scape; spmth – spermatheca; st – sternite; tg – tergite.

*Adelphomyiaacicularisbidens Savchenko, 1983 (Figs 4–8)

**Figures 9–11.**
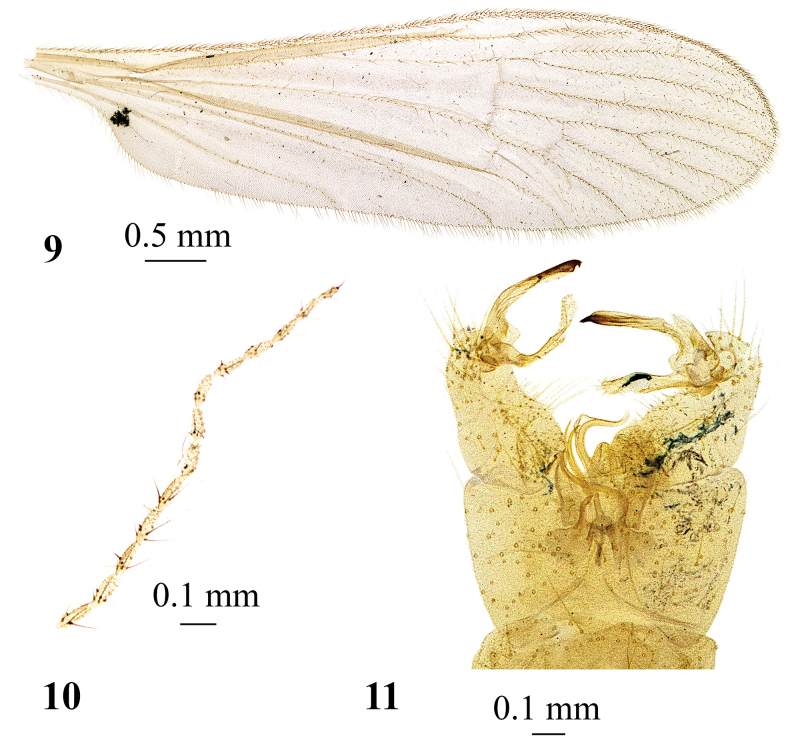
*Adelphomyiabiacus* (Alexander, 1954), male, holotype **9** wing **10** antenna **11** genitalia, dorsal view.

*Adelphomyiabiacus* (Alexander, 1954) (Figs 9–11)

**Figures 12–14.**
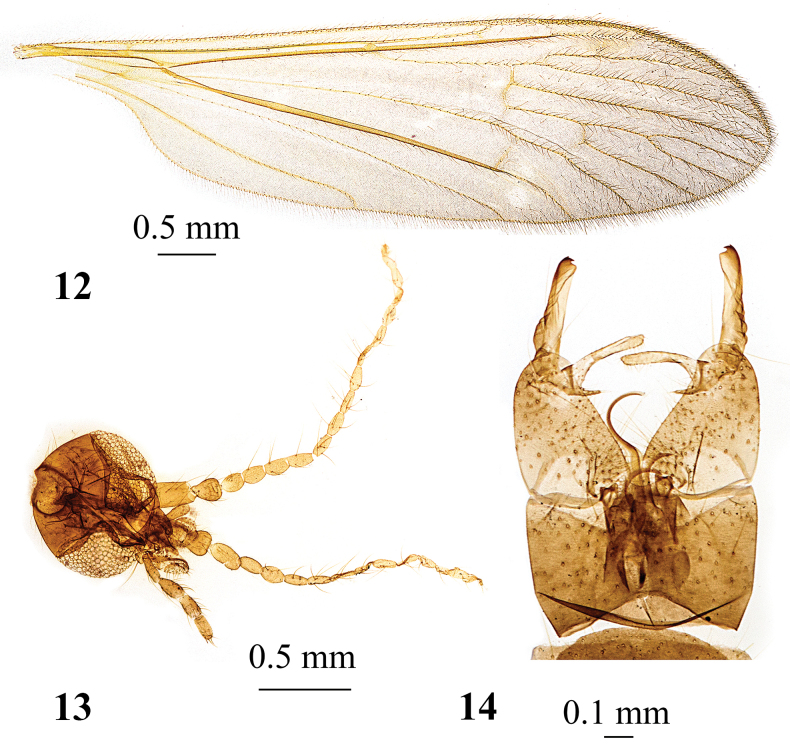
*Adelphomyiabreviramus* (Alexander, 1924), male **12** wing, holotype **13** head, dorsal view **14** genitalia, dorsal view, holotype.

*Adelphomyiabreviramus* (Alexander, 1924) (Figs 12–14)

**Figures 15, 16.**
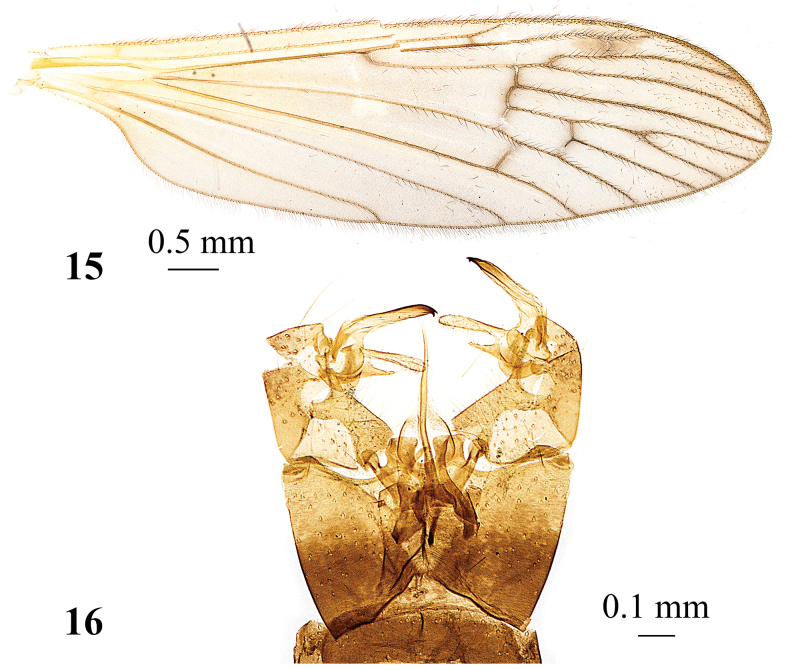
*Adelphomyiacaesiella* (Alexander, 1929), male **15** wing **16** genitalia, dorsal view.

*Adelphomyiacaesiella* (Alexander, 1929) (Figs 15, 16)

**Figures 17–21.**
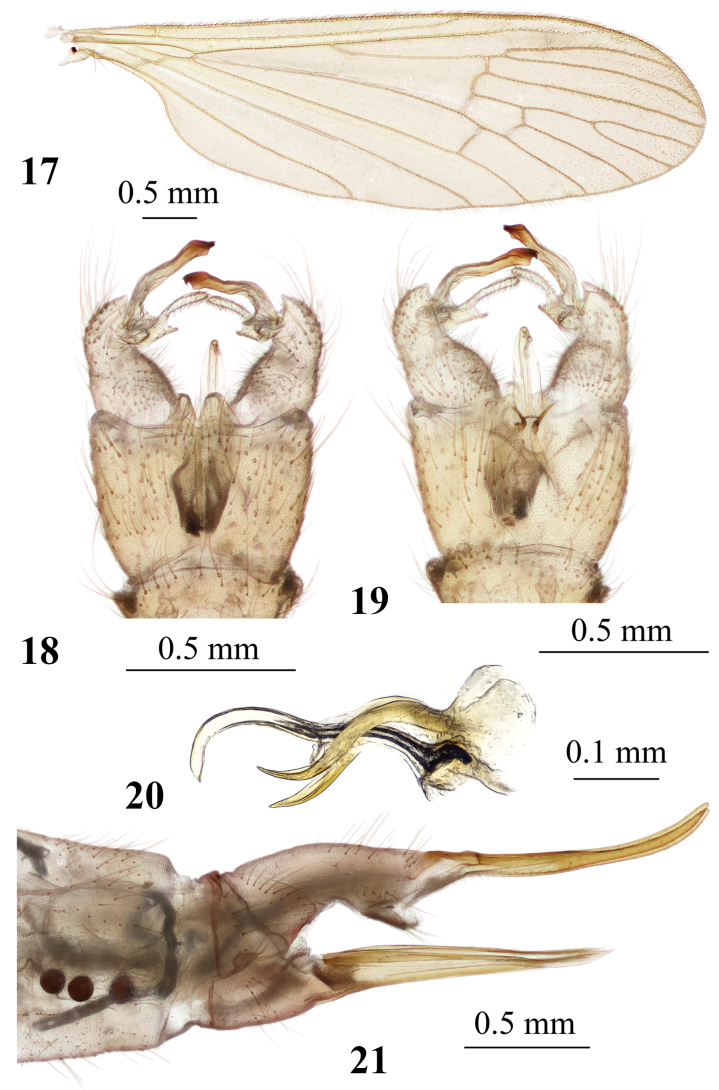
*Adelphomyiaflavella* (Alexander, 1920) **17** male wing **18** male genitalia, dorsal view **19** male genitalia, ventral view **20** aedeagal complex, lateral view **21** ovipositor, lateral view.

**Figures 22, 23.**
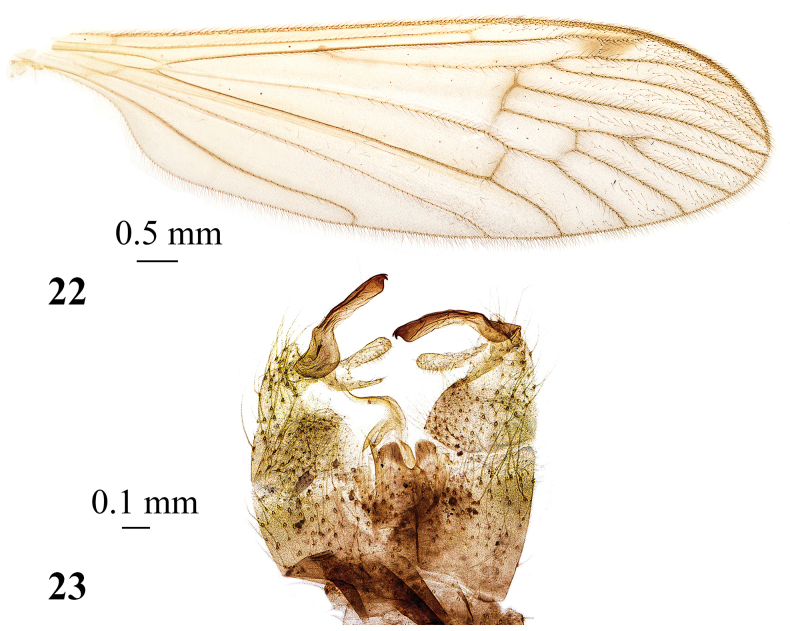
*Adelphomyiamacrotrichiata* (Alexander, 1923), male, holotype **22** wing **23** genitalia, dorsal view.

*Adelphomyiaflavella (Alexander, 1920) (Figs 17–21)

(*)*Adelphomyiamacrotrichiata* (Alexander, 1923) (record from North Korea based on misidentification) (Figs 22, 23)

**Figures 24–26.**
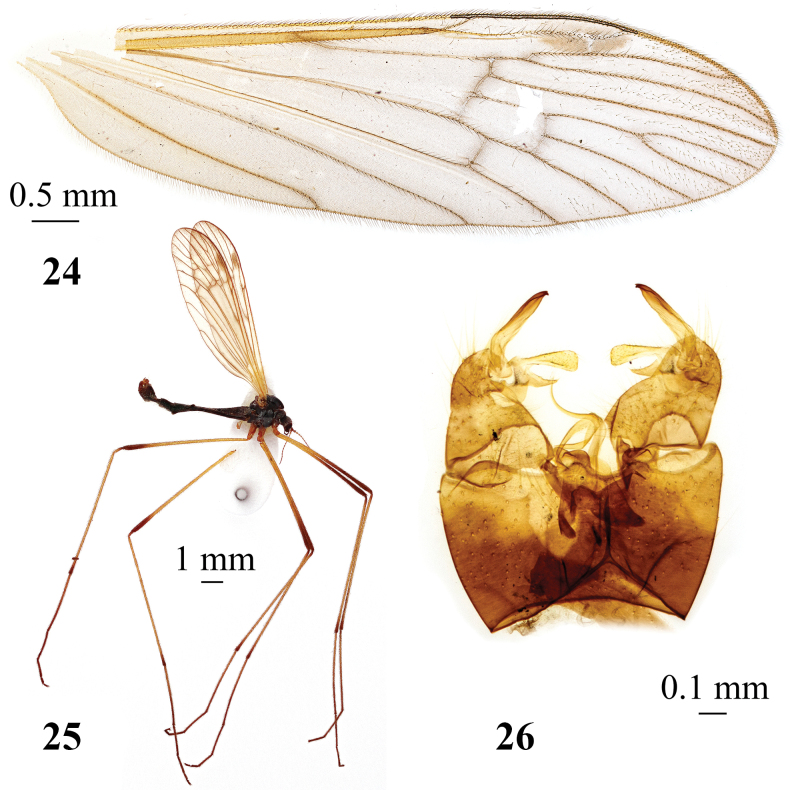
*Adelphomyiapilifer* (Alexander, 1919), male **24** wing, paratype **25** general view **26** genitalia, dorsal view, paratype.

*Adelphomyiapilifer* (Alexander, 1919) (Figs 24–26)

**Figures 27, 28.**
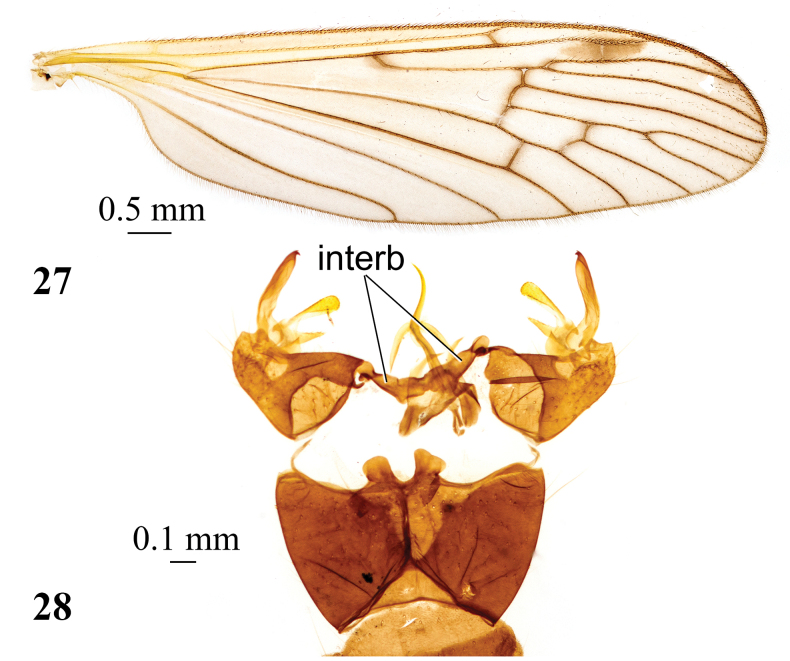
*Adelphomyiaprionolaboides* (Alexander, 1934), male **27** wing **28** genitalia, dorsal view. Abbreviation: interb – interbases.

*Adelphomyiaprionolaboides* (Alexander, 1934) (Figs 27, 28)

**Figures 29–33.**
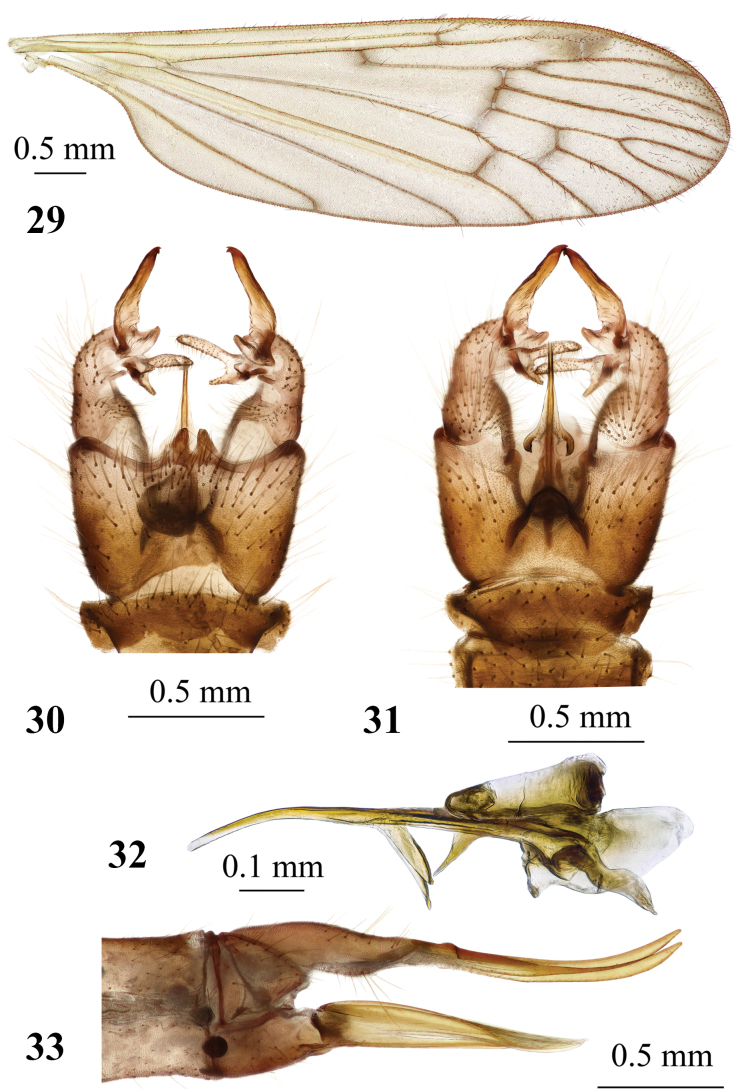
*Adelphomyiapunctum* (Meigen, 1818) **29** male wing **30** male genitalia, dorsal view **31** male genitalia, ventral view **32** aedeagal complex, lateral view **33** ovipositor, lateral view.

*Adelphomyiapunctum (Meigen, 1818) (Figs 29–33)

**Figures 34, 35.**
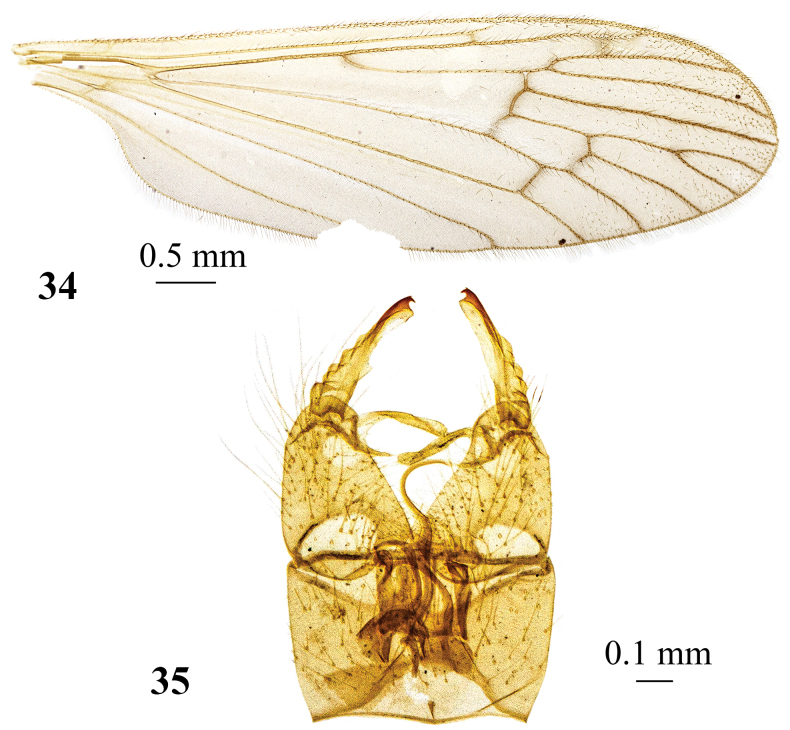
*Adelphomyiasaitamae* (Alexander, 1920), male **34** wing **35** genitalia, dorsal view.

*Adelphomyiasaitamae* (Alexander, 1920) (Figs 34, 35)

**Figures 36, 37.**
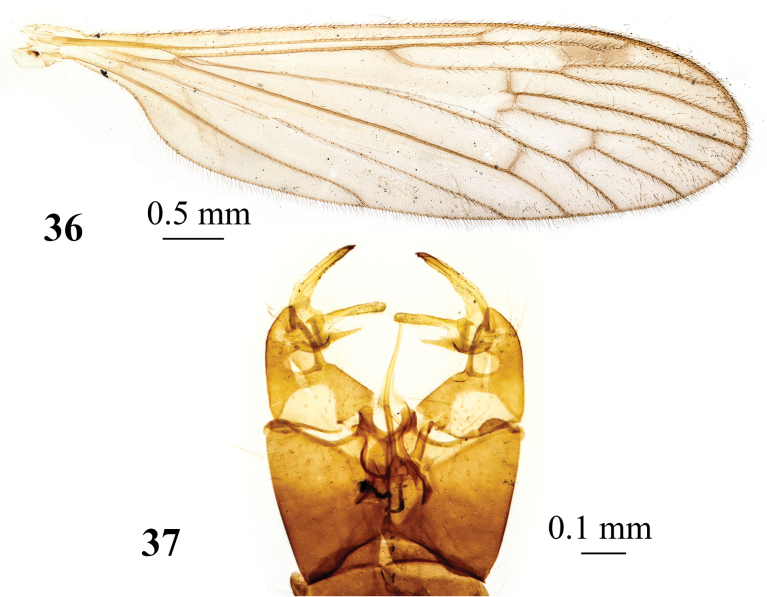
*Adelphomyiasatsumicola* (Alexander, 1930), male, holotype **36** wing **37** genitalia, dorsal view.

*Adelphomyiasatsumicola* (Alexander, 1930) (Figs 36, 37)

**Figures 38, 39.**
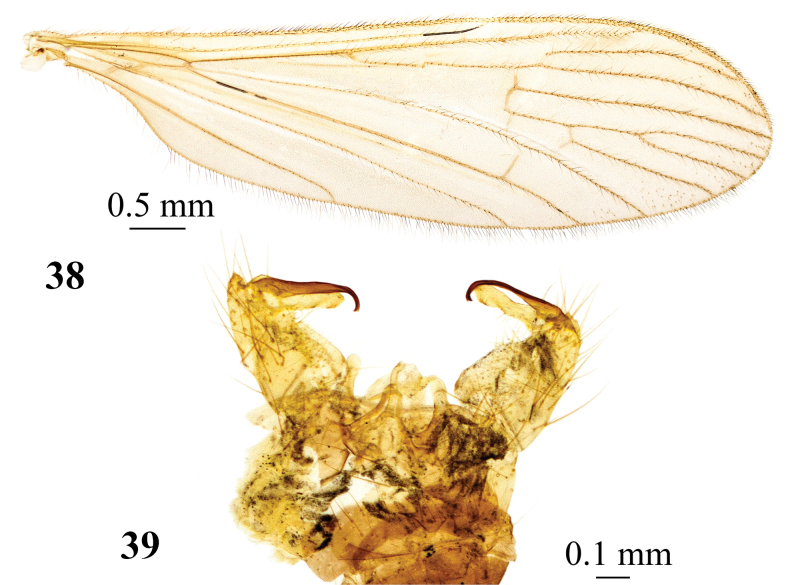
*Adelphomyiasimplicistyla* (Alexander, 1940b), male, holotype **38** wing **39** genitalia, dorsal view.

*Adelphomyiasimplicistyla* (Alexander, 1940b) (Figs 38, 39)

**Figures 40–43.**
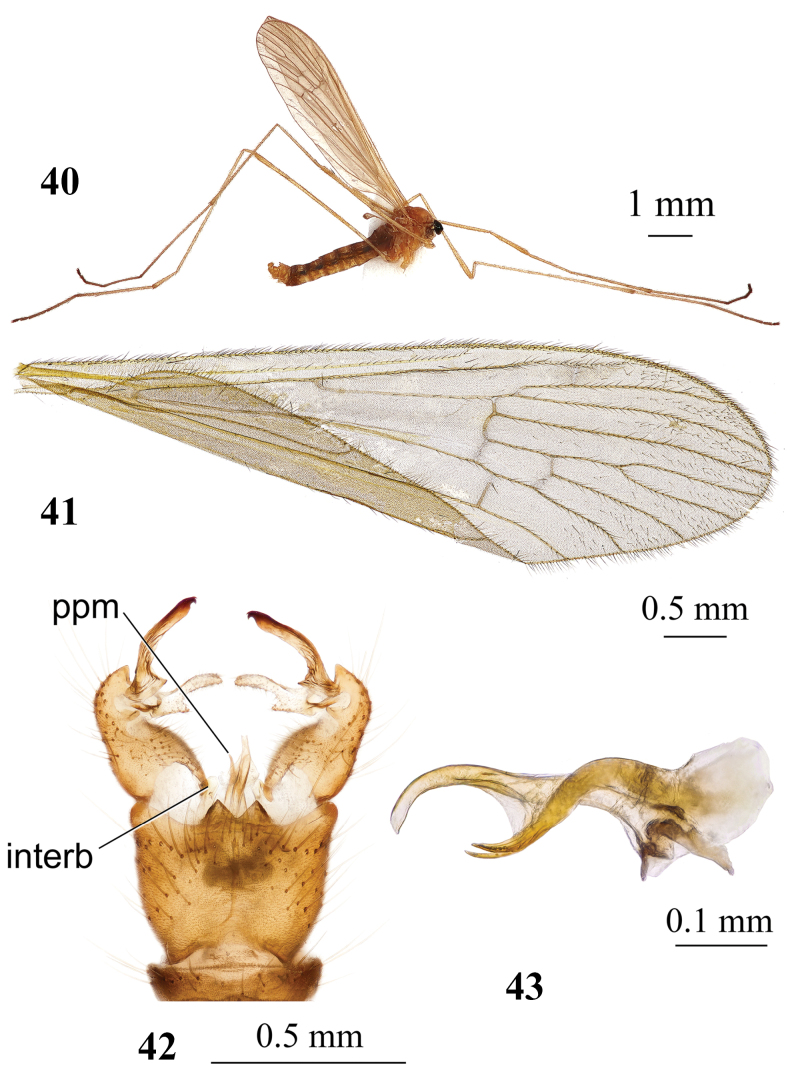
*Adelphomyiajejuana* Podenas, sp. nov., holotype, male **40** general view **41** wing **42** genitalia, dorsal view **43** aedeagal complex, lateral view. Abbreviations: interb – interbase; ppm – posterior part of paramere.

**Figures 44–46.**
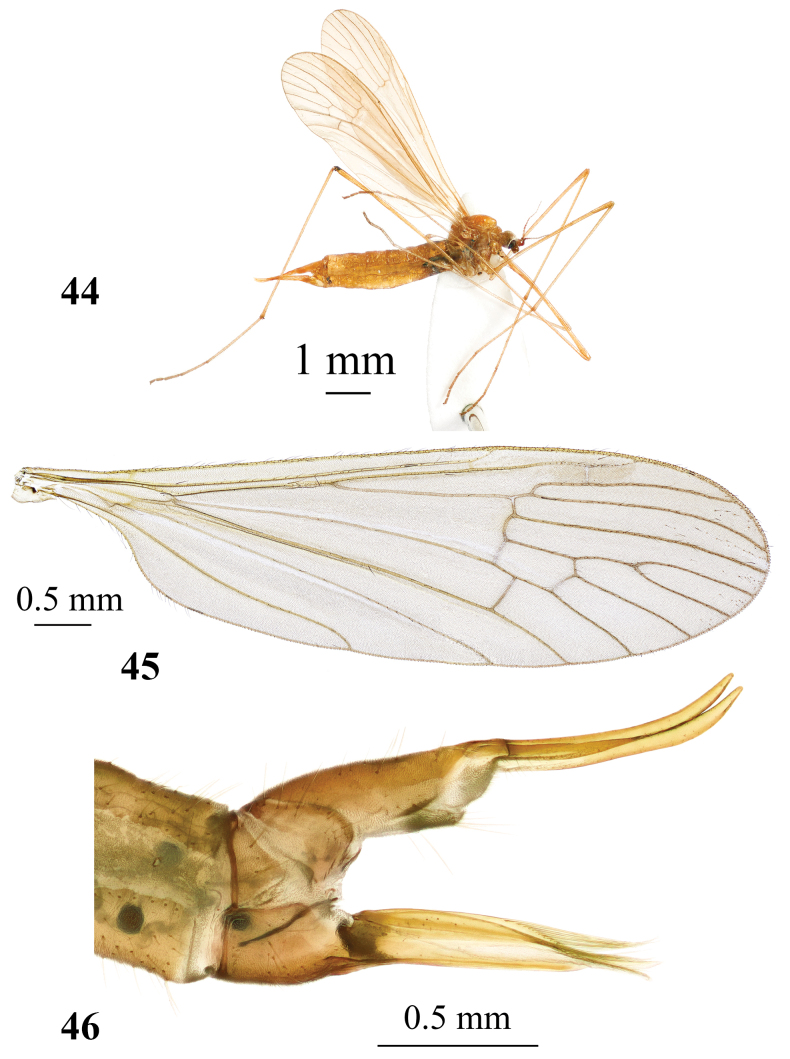
*Adelphomyiajejuana* Podenas, sp. nov., paratype, female **44** general view **45** wing **46** ovipositor, lateral view.

*Adelphomyiajejuana Podenas, sp. nov. (Figs 40–46)

##### ﻿Key to Palaearctic species of the genus *Adelphomyia*

**Table:** 

1	Main body colour, including thorax and abdomen, dark (dark grey, brown, or black) (Fig. 25)	**2**
–	Main body colour, including thorax and abdomen, pale (yellow, brownish yellow or pale brown) (Figs 40, 44)	**8**
2	Entire mesonotum dull grey or brown	**3**
–	Mesonotum polished black anteriorly	***A.pilifer* (Alexander, 1919)**
3	Dark areas surrounding cross-veins restricted but evident (Figs 1, 5, 15, 27)	**4**
–	Dark areas surrounding cross-veins missing (Fig. 12) or indistinct (Fig. 36)	**7**
4	Head dark grey, thorax dark grey, abdomen brown, femur obscure yellow	**5**
–	Head pale grey, thorax grey or brownish grey with black stripes, abdomen black, femur yellow with widely darkened tip	**6**
5	Medial lobes of epandrium parallel-sided, notch between them U-shaped (Fig. 3)	***A.acicularisacicularis* (Alexander, 1954)**
–	Medial lobes of epandrium diverging distally, notch between them V-shaped (Fig. 6)	***A.acicularisbidens* Savchenko, 1983**
6	Thorax grey, antenna pale brown	***A.caesiella* (Alexander, 1929)**
–	Thorax brownish grey with black stripes, antenna brown to dark brown	***A.prionolaboides* (Alexander, 1934)**
7	Thorax reddish brown, abdomen dark brown, femur obscure yellow, head dark grey, antenna pale brown (Fig. 13), macrotrichiae covering nearly entire cell *r_3_* (Fig. 12), medial lobes of epandrium diverging distally, notch between them shallowly U-shaped (Fig. 14)	***A.breviramus* (Alexander, 1924)**
–	Thorax grey, abdomen brownish grey, femur yellow with narrowly dark tip, head grey, antenna dark brown, macrotrichiae covering only outer end of cell *r_3_* (Fig. 36), medial lobes of male epandrium parallel-sided, notch between them widely U-shaped (Fig. 37)	***A.satsumicola* (Alexander, 1930)**
8	Complete body, including thorax, abdomen, head, antennae (Fig. 10), legs, and wings (Fig. 9), yellow or pale yellow	***A.biacus* (Alexander, 1954)**
–	Thorax or abdomen grey or brownish (Figs 40, 44)	**9**
9	Wing pattern indistinct or missing (Figs 17, 22, 38, 41, 45)	**10**
–	Wing pattern restricted but evident (Figs 29, 34)	**13**
10	Thorax brown. Macrotrichiae covering outer ends of cells *r_2_* to *m_4_* (Fig. 38)	***A.simplicistyla* (Alexander, 1940b)**
–	Thorax brownish yellow. Macrotrichiae covering nearly entire or only outer ends of cells *r_2_* to *m_4_* (Figs 17, 22, 41, 45)	**11**
11	Abdominal tergites brownish yellow. Notch between medial lobes of male epandrium V-shaped (Figs 18, 42)	**12**
–	Abdominal tergites brown. Notch between medial lobes of male epandrium U-shaped (Fig. 23)	***A.macrotrichiata* (Alexander, 1923)**
12	Head brownish yellow with yellow antennae. Macrotrichiae covering nearly entire cells *r_2_* to *m_4_* (Fig. 17)	***A.flavella* (Alexander, 1920)**
–	Head greyish brown with pale brown antennae. Macrotrichiae covering only outer ends of cells *r_2_* to *m_4_* (Figs 41, 45)	***A.jejuana* Podenas, sp. nov.**
13	Head brownish yellow, tips of femora not darkened. Medial lobes of male epandrium diverging distally, notch between them V-shaped (Fig. 35)	***A.saitamae* (Alexander, 1920)**
–	Head grey, tips of femora narrowly darkened. Medial lobes of male epandrium acute, notch between them narrow, parallel-sided (Fig. 30)	***A.punctum* (Meigen, 1818)**

### ﻿ Adelphomyia acicularis bidens

Taxon classificationAnimaliaDipteraLimoniidae

Savchenko, 1983

C9DF43B8-FDFB-52F4-9CC7-0D8F306B499F

Figs 4–8

Adelphomyia acicularis bidens Savchenko, 1983: 53.

#### Examined material.

**Figures 47–50.**
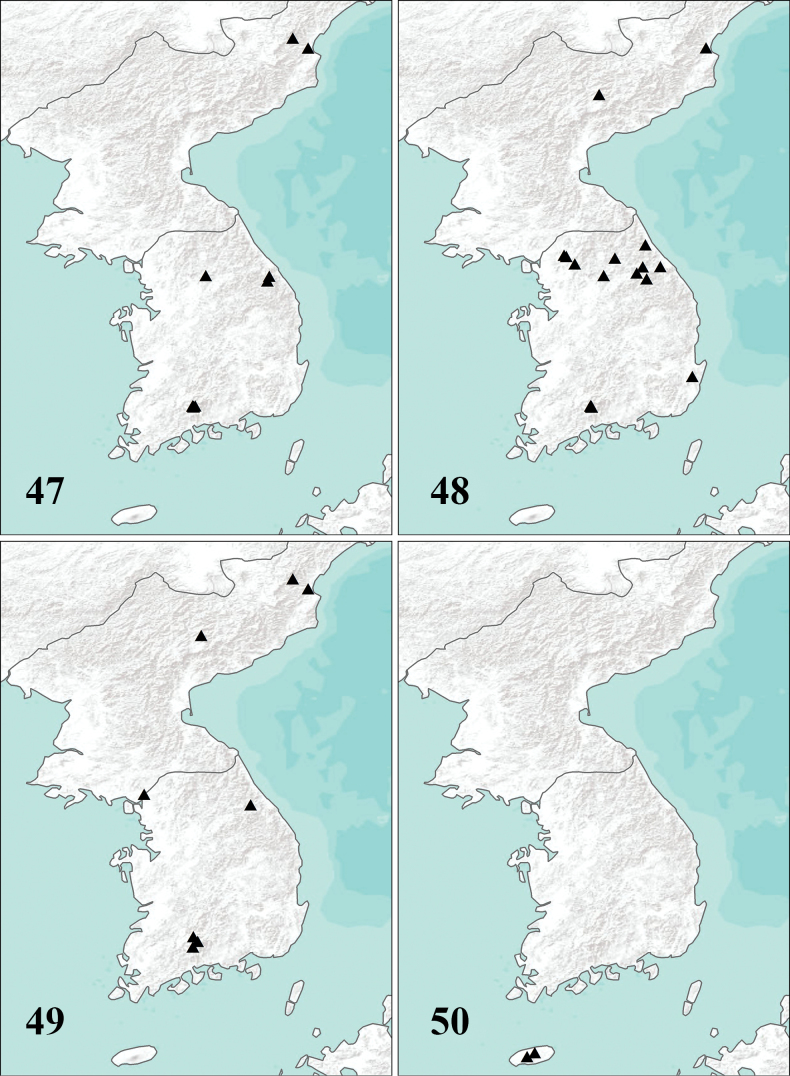
Sampling localities of Korean *Adelphomyia***47***A.acicularisbidens* Savchenko, 1983 **48***A.flavella* (Alexander, 1920) **49***A.punctum* (Meigen, 1818) **50***A.jejuana* Podenas, sp. nov.

(Fig. 47) **North Korea** • 2 ♂ (pinned); Ompo; alt. 122 m; 29 May 1938; A. M. Yankovsky leg.; USNM • 3 ♂ (pinned); Seren Mts.; alt. 1067 m; 22 June 1938; A. M. Yankovsky leg.; USNM; **South Korea** • 1 ♂ (in ethanol); Gangwon-do, Pyeongchang-gun, Daegwallyeong-myeon, Yongsan-ri, Mt. Balwangsan; 19 July 2008; J. D. Yeo, M. J. Jeon and K. G. Kim leg.; Malaise trap; NIBR • 1 ♀ (in ethanol); Gangwon-do, Jeongseon-gun, Imgye-myeon, Dojeon-ri; 37.53583°N, 128.90278°E; alt. 762 m; 24 May – 23 June 2011; H.-W. Byun et al. leg.; Malaise trap; NIBR • 1 ♂ (in ethanol); Gyeongsangnam-do, Hadong-gun, Hwagae-myeon, Beomwang-ri; 35.27360°N, 127.61121°E; alt. 369 m; 8 May 2013 (2); S. Podenas leg.; NIBR • 1 ♂ (in ethanol); Jeollanam-do, Gurye-gun, Toji-myeon, Naedong-ri; 35.26580°N, 127.58128°E; alt. 378 m; 10 May 2013; S. Podenas leg.; NIBR • 1 ♂ (in ethanol); Jeollanam-do, Gurye-gun, Toji-myeon, Naedong-ri; 35.26580°N, 127.58128°E; alt. 378 m; 11 May 2013; S. Podenas leg.; at light; NIBR • 1 ♂ (pinned); Jeollanam-do, Gurye-gun, Toji-myeon, Naeseo-ri, Piagol valley; 35.25825°N, 127.58208°E; alt. 310 m; 26 April 2015 (2); S. Podenas leg.; NIBR • 2 ♂ (in ethanol); Jeollanam-do, Gurye-gun, Toji-myeon, Naeseo-ri, Jirisan National Park, Piagol valley; 35.27448°N, 127.56378°E; alt. 593 m; 1 May 2015 (1); S. Podenas leg.; at light; NIBR • 1 ♂ (pinned); Jeollanam-do, Gurye-gun, Toji-myeon, Naeseo-ri, Jirisan National Park, Piagol valley; 35.27177°N, 127.57146°E; alt. 490 m; 2 May 2015 (1); S. Podenas leg.; NIBR • 2 ♂ (in ethanol); Jeollanam-do, Gurye-gun, Toji-myeon, Naeseo-ri, Jirisan National Park, Piagol valley; 35.25825°N, 127.58208°E; alt. 310 m; 2 May 2015 (2); S. Podenas leg.; NIBR • 1 ♂ (pinned); Jeollanam-do, Gurye-gun, Toji-myeon, Naeseo-ri, Jirisan National Park, Piagol valley; 35.26590°N, 127.58096°E; alt. 446 m; 2 May 2015 (4); S. Podenas leg.; at light; NIBR • 2 ♂ (in ethanol); Jeollanam-do, Gurye-gun, Toji-myeon, Naeseo-ri, Jirisan National Park, Piagol valley; 35.26590°N, 127.58096°E; alt. 446 m; 3 May 2015 (3); S. Podenas leg.; at light; NIBR • 1 ♂ (in ethanol); Jeollanam-do, Gurye-gun, Toji-myeon, Naeseo-ri, Jirisan National Park, Piagol valley; 35.26590°N, 127.58096°E; alt. 446 m; 27 June 2015 (2); S. Podenas leg.; at light; NIBR • 2 ♂ (in ethanol); Gyeonggi-do, Yangpyeong, Cheongun-myeon, Dowon-ri; 37.54507°N, 127.79483°E; alt. 224 m; 28 May 2017; S. Podenas leg.; at light; NIBR.

#### Redescription.

General body colouration brown to dark brown densely covered with grey pruinosity. Body length of male 5.6–8.4 mm, of female 8.0 mm. Wing length of male 6.3–8.7 mm, of female 8.4 mm.

***Head.*** Dark brown, dusted with grey, pale grey pruinose frontally and along eye margin. Eyes widely separated in both sexes, distance between them at base of antennae exceeds length of scape. Antenna (Fig. 4) 1.7–2.8 mm long in male, 1.7 mm in female, extending beyond wing base if bent backward. Scape greyish brown, elongate, nearly cylindrical, 2× as long as pedicel, pedicel pear-shaped. Flagellum yellow basally, slightly darkened distally. Flagellomeres elongate, longest at middle, apical flagellomere slightly smaller than penultimate. Verticils brownish, longest verticils ~ 1.5× as long as respective segments. Rostrum dark brown dusted with grey, palpus and labellum dark brown.

***Thorax.*** Cervical sclerites brown to dark brown. Pronotum pale brown to dark brown, depending on specimen, covered with sparse erect long yellowish setae dorsally. Presutural scutum brown, dusted with grey, without stripes or with indistinct darker median stripes on the anterior half. Tubercular pit small, polished-brown at frontal margin of sclerite, prescutal pit distinct, polished-brown, surrounded by grey area. Scutal lobe and scutellum brown, dusted with grey, area between scutal lobes pale brown, mediotergite brown, dusted with grey along middle, yellowish brown laterally. Pleuron uniformly brown, densely covered with brown pruinosity. Wing (Fig. 5) brownish. Stigma elongate, from pale brown to brown. Cross-veins and branching points of veins narrowly surrounded by indistinct darker areas in some specimens, in other specimens darker areas missing. Veins brownish, yellowish at wing base. Macrotrichiae more abundant in radial cells and cell *m_1_*, also present in other marginal cells along postero-apical wing margin, few macrotrichiae present also in cell *cua* at wing margin. Venation: *Sc* long, reaching slightly before branching point of *Rs*, *sc-r* less than its own length from tip of *Sc. Rs* long, slightly arched at base. Free end of *R_1_* longitudinal, *R_2_* transverse, indistinct, 3× its own length from tip of *R_1_*, *R_3_*, and *R_4_* slightly diverging towards wing margin, cell *r_3_* with short stem. Cross-vein *r-m* distinct, at base of discal cell. Discal cell slightly more than 2× as long as wide. Cross-vein *m-cu* slightly before middle of discal cell. Anal vein slightly arched at apex, reaching wing margin at the level of *Rs* base. Anal angle widely rounded. Length of male halter 1.0–1.5 mm, of female 1.2 mm. Halter pale yellowish, knob slightly infuscate. Coxae yellow, only fore coxa brownish at base. Trochanters pale yellow. Femora yellow with indistinctly darkened apices, tibiae yellow with darker distal ends. Basal tarsomeres pale brown, remaining tarsomeres dark brown. Male femur I: 4.1–5.2 mm long, II: 4.0–5.4 mm, III: 4.5–6.0 mm, tibia I: 5.3–6.5 mm, II: 4.8–5.9 mm, III: 6.0–6.5 mm, tarsus I: 5.1–6.2 mm, II: 5.0–5.6 mm, III: 4.6–5.0 mm. Female femur II: 4.7 mm long, tibia II: 4.7 mm, tarsus II: 4.3 mm. Claw simple, without spines.

***Abdomen.*** Tergites brown, dusted with grey, sternites yellowish brown, paler at base of abdomen. Male terminalia (Fig. 6) yellow. Ninth tergite with two diverging triangle-shaped lobes at the middle of posterior margin and wide V-shaped indentation between them. Gonocoxite elongate, wider at base, narrower beyond middle, without additional lobe. Outer gonostylus with long, narrow, slightly arched outer branch that has few transverse ridges on basal half and small triangle-shaped lobe at base. Outer branch with sclerotised distal part and blackened apex, two small apical hook-shaped teeth curved medially. Inner gonostylus large, fleshy, setose, two-branched. Outer branch long, narrow, inner branch short, very narrow, reaching to ~ 1/3 of outer branch. Aedeagus (Fig. 7) strongly curved ventrally at ~ 2/3 length, distal part at ~ 90^0^ angle to basal part (clearly visible in lateral view). Paramere darkened, posteriorly long, narrow, rod-shaped, nearly as long as aedeagus, strongly curved at ~ 1/6 length; basal part extends parallel to main body axis, distal part directed exactly downwards. Interbase nearly oblong with tip rounded. Ovipositor (Fig. 8) brownish yellow. Cercus long, narrow, apical part slightly raised upwards, apex obtuse. Hypogynial valve long, straight, wide at base, apical part distinctly narrower, apex reaching beyond middle of cercus, dorsal margin at ~ 1/3 length from tip with long straight setae.

#### Elevation range.

From 100 m to 1100 m.

#### Period of activity.

From end of April through middle of July.

#### Habitat.

Slopes to small mountainous streams densely covered with deciduous trees and shrubs, moss tufts on rocks. Species is attracted to light.

#### General distribution.

The nominotypical subspecies is known only from Shikoku island, Japan, subspecies *A.acicularisbidens* only from southern part of the Far East of Russia. Species and subspecies recorded from the Korean Peninsula for the first time.

### ﻿ Adelphomyia flavella

Taxon classificationAnimaliaDipteraLimoniidae

(Alexander, 1920)

1C239A37-E3D8-50CA-A785-B13A3590FFE6

Figs 17–21

Limnophila (Lasiomastix) flavella Alexander, 1920: 12.
Adelphomyia flavella : Oosterbroek 2024.

#### Type material examined.

**Japan • Paratype** ♂ (as *Limnophillaflavella*), wing and genitalia slide-mounted; Tokio; 5 May 1919; R. Takahashi leg.; USNM.

#### Other examined material.

(Fig. 48) **North Korea** • 6 ♀ (pinned); Ompo; alt. 37 m; 15 June 1937; A. M. Yankovsky leg.; USNM • 1 ♀ (pinned); Ompo; alt. 122 m; 3 June 1938; A. M. Yankovsky leg.; USNM • 2 ♀ (pinned); Ompo; alt. 107 m; 11 June 1938; A. M. Yankovsky leg.; USNM • 3 ♂ (pinned); Kankyo Nando, Puksu Pyaksan; alt. 1219 m; 9 June 1939; A. M. Yankovsky leg.; USNM • 1 ♂ (pinned); Kankyo Nando, Puksu Pyaksan; alt. 1280 m; 6 June 1939; A. M. Yankovsky leg.; USNM • 1 ♂ (pinned); Kankyo Nando, Puksu Pyaksan; alt. 1433 m; 6 June 1939; A. M. Yankovsky leg.; USNM • 1 ♂ (pinned); Kankyo Nando, Puksu Pyaksan; alt. 1463 m; 6 June 1939; A. M. Yankovsky leg.; USNM • 1 ♂, 1 ♀ (pinned); Kankyo Nando, Puksu Pyaksan; alt. 1676 m; 8 June 1939; A. M. Yankovsky leg.; USNM; **South Korea** • 1 ♂, 5 ♀ (pinned); #8, Central National Forest, 29 km NE Seoul; alt. 122–152 m; 28 May 1954; George W. Byers leg.; 2 ♀ USNM; 1 ♂, 3 ♀ SMEK • 3 ♂, 1 ♀ (pinned); #9, Central National Forest, 29 km NE Seoul; alt. 122–152 m; 29 May 1954; George W. Byers leg.; SMEK • 1 ♂ (pinned); #12, Hwy. #20, 13 km SW Kangnung; 37.70000°N, 128.78333°E; alt. 587 m; 8 June 1954; George W. Byers leg.; SMEK • 1 ♂ (pinned); #17, Central National Forest, 29 km NE Seoul; alt. 107–152 m; 20 June 1954; George W. Byers leg; SMEK • 1 ♀ (in ethanol); Gangwon-do, Pyeongchang-gun, Yongpyeong-myeon, Nodong-ri, Mt. Gyebangsan; 19 July – 12 August 2008; H. Y. Seo & K. G. Kim leg.; Malaise trap; NIBR • 1 ♂ (in ethanol); Haanmi-ri, Daehwa-myeon, Pyeongchang-gun, Gangwon-do, Mt. Gariwangsan; 37.45028°N, 128.50306°E; 13 May – 3 June 2009; W. Y. Choi et al. leg.; NIBR • 1 ♂ (in ethanol); Gangwon-do, Pyeongchang-gun, Jinbu-myeon, Jangjeon-ri, Mt. Gariwangsan; 37.48778°N, 128.54528°E; alt. 693 m; 4 –17 June 2009; J. D. Yeo, J. D. Yoon leg.; Malaise trap; NIBR • 1 ♀ (in ethanol); Jeollanam-do, Gurye, Toji-myeon, Naedong-ri; 35.26580°N, 127.58128°E; alt. 378 m; 11 May 2013; S. Podenas leg.; at light; NIBR • 1 ♂ (in ethanol); Jeollanam-do, Gurye-gun, Toji-myeon, Naedong-ri; 35.26580°N, 127.58128°E; alt. 378 m; 12 May 2013; S. Podenas leg.; NIBR • 4 ♂, 1 ♀ (in ethanol); Jeollanam-do, Gurye-gun, Toji-myeon, Naeseo-ri, Piagol valley; 35.25257°N, 127.58981°E; alt. 304 m; 29 April 2015 (1); S. Podenas leg.; NIBR • 3 ♂, 2 ♀ (in ethanol); Jeollanam-do, Gurye-gun, Toji-myeon, Naeseo-ri, Piagol valley; 35.25825°N, 127.58208°E; alt. 310 m; 2 May 2015 (2); S. Podenas leg.; NIBR • 2 ♂ (in ethanol); Jeollanam-do, Gurye-gun, Toji-myeon, Naeseo-ri, Piagol valley; 35.26590°N, 127.58096°E; alt. 446 m; 3 May 2015 (3); S. Podenas leg.; at light; NIBR • 1 ♀ (in ethanol); Jeollanam-do, Gurye-gun, Toji-myeon, Naeseo-ri, Piagol valley; 35.27448°N, 127.56378°E; alt. 593 m; 1 July 2015 (1); V. Podeniene leg.; NIBR • 1 ♂ (in ethanol); Gangwon-do, Yangyang-gun, Seo-myeon, Garapi-ri; 38.07933°N, 128.52042°E; alt. 160 m; 7 July 2015 (1); S. Kim, S. Podenas leg.; NIBR • 1 ♀ (in ethanol); Gangwon-do, Hongcheon-gun, Duchon-myeon, Cheonhyeon-ri, near Mt. Garisan; 37.84840°N, 127.98879°E; alt. 304 m; 8 July 2015 (3); S. Kim, S. Podenas leg.; NIBR • 2 ♂, 2 ♀ (in ethanol); Gyeongsangbuk-do, Gyeongju-si, Jinhyeon-dong, Tohamsan (Mt.), 1.3 km southeast from Seokgulam; 35.78797°N, 129.33919°E; alt. 297 m; 27 May 2016; S. Podenas, H.M. Baek leg.; NIBR • 1 ♀ (in ethanol); Jeollanam-do, Gurye-gun, Toji-myeon, Naeseo-ri, Piagol valley; 35.27177°N, 127.57146°E; alt. 490 m; 3 June 2016 (2); S. Podenas leg.; NIBR • 1 ♂ (in ethanol); Jeollanam-do, Gurye-gun, Toji-myeon, Naeseo-ri, Piagol valley; 35.26586°N, 127.58090°E; alt. 448 m; 3 June 2016 (4); S. Podenas leg.; at light; NIBR • 2 ♂ (in ethanol); Gyeonggi-do, Yangpyeong, Cheongun-myeon, Dowon-ri; 37.54507°N, 127.79483°E; alt. 224 m; 28 May 2017 (1); S. Podenas leg.; with net and at light; NIBR • 1 ♂ (in ethanol); Gyeonggi-do, Dongducheon, Tapdong-dong, Casey; 37.87845°N, 127.14566°E; alt. 503 m; 20 June 2017; T. A. Klein, H.-C. Kim leg.; NJ trap; NIBR • 2 ♂ (in ethanol); Gyeonggi-do, Dongducheon, Tapdong-dong, Casey; 37.87845°N, 127.14566°E; alt. 503 m; 26 June 2017; T. A. Klein, H.-C. Kim leg.; NJ trap; NIBR • 1 ♂ (in ethanol); Gyeonggi-do, Dongducheon-si, Gwangam-dong, Hovey; 37.90044°N, 127.10319°E; alt. 353 m; 26 June 2017; T. A. Klein, H.-C. Kim leg.; NJ trap; NIBR • 1 ♂, 1 ♀ (pinned); Jeollanam-do, Gurye-gun, Toji-myeon, Naeseo-ri, Piagol valley; 35.27333°N, 127.56924°E; alt. 546 m; 25 June 2019 (1); S. Podenas leg.; NIBR.

#### Redescription.

General body colouration brownish yellow. Body length of male 4.2–6.7 mm, of female 5.3–7.9 mm. Wing length of male 5.5–7.3 mm, of female 5.7–7.7 mm.

***Head.*** Brownish greyish yellow, pale grey pruinose frontally and along eye margin. Eyes widely separated in both sexes, distance between them at base of antennae exceeds lengths of scape and pedicel combined. Antenna 1.2–2.1 mm long in male, 0.9–1.7 mm in female, reaching to the wing base if bent backward. Both basal antennomeres yellow, scape slightly dusted with grey basally, elongate, nearly cylindrical, 2× as long as pedicel, pedicel pear-shaped. Flagellum yellow basally, slightly darkened distally. Flagellomeres elongate, longest at middle, apical flagellomere smaller than penultimate. Verticils brownish, longest verticils approximately as long as respective segments. Rostrum pale brown, palpus darker brown, mouth parts pale brown.

***Thorax.*** Cervical sclerites yellow. Pronotum pale brown dorsally, yellow laterally, covered with long, sparse, erect, yellow setae dorsally. Mesonotal prescutum uniformly brownish yellow, without stripes, sparsely dusted with grey. Tubercular pit indistinct, pseudosutural fovea concolourous with presutural scutum. Scutal lobes and scutellum brownish yellow, mediotergite yellow to brownish yellow. Pleuron yellow dorsally, pale yellow ventrally, sparsely dusted with greyish yellow. Wing (Fig. 17) semi-translucent, pale yellow, without darker areas except stigma. Stigma indistinct, brownish. Veins pale brown, yellowish at wing base and in costal area. Macrotrichiae more abundant in radial cells and cell *m_1_*, also present in other marginal cells along postero-apical wing margin, few macrotrichiae present in cell *cua* at wing margin. Venation: *Sc* long, reaching slightly before branching point of *Rs*, *sc-r* ~ 3× its own length from tip of *Sc. Rs* long, slightly arched at base. Free end of *R_1_* longitudinal, *R_2_* transverse, indistinct, 3.3× its own length from tip of *R_1_*, *R_3_*, and *R_4_* slightly arched and diverging towards wing margin, cell *r_3_* with short stem. Cross-vein *r-m* distinct, at base of discal cell. Discal cell 1.8× as long as wide. Cross-vein *m-cu* at middle of discal cell. Anal vein slightly arched at wing margin, ending beyond base of *Rs.* Anal angle widely rounded. Length of male halter 0.8–1.2 mm, of female 0.8–1.6 mm. Halter pale, knob slightly infuscate. Coxae and trochanters yellow to pale yellow. Femur and tibia yellow, tibia slightly infuscate at apex. Basal tarsomere pale brown, remaining tarsomeres dark brown. Male femur I: 3.6–4.3 mm long, II: 4.0–4.8 mm, III: 3.9–5.0 mm, tibia I: 4.5–5.4 mm, II: 3.9–4.9 mm, III: 4.0–5.2 mm, tarsus I: 4.6–5.5 mm, II: 4.2–5.0 mm, III: 3.3–4.2 mm. Female femur I: 3.3–4.0 mm long, II: 3.1–4.4 mm, III: 3.4–4.9 mm, tibia I: 3.9–4.4 mm, II: 3.0–4.0 mm, III: 3.2–4.6 mm, tarsus I: 3.6–4.1 mm, II: 3.2–5.0 mm, III: 3.0–3.6 mm. Claw simple, without spines.

***Abdomen.*** Tergites brownish yellow, sternites yellow. Male terminalia (Figs 18, 19) yellow. Ninth tergite with two triangle-shaped lobes at the middle of posterior margin and V-shaped indentation between them. Gonocoxite elongate, distinctly wider at base, narrower beyond middle, without additional lobe. Outer gonostylus with long, narrow, slightly sinuous outer branch and small triangle-shaped lobe at base. Outer branch with sclerotised distal part and blackened apex, two small apical hook-shaped teeth curved medially. Inner gonostylus large, fleshy, setose, two-branched. Outer branch long and narrow, inner branch short, reaching to ~ 1/3 of outer branch. Aedeagus (Fig. 20) strongly curved at ~ 2/3 length, distal part at ~ 90 ^0^ angle to basal part (clearly visible in lateral view). Paramere posteriorly short, narrow, rod-shaped with darkened distal part, reaching just slightly beyond base of gonocoxite (best visible in ventral view). Interbase with tip rounded. Ovipositor (Fig. 21) pale yellow. Cercus very long, narrow, distal part slightly raised upwards. Hypogynial valve long, wedge-shaped, pointed apex reaching slightly beyond middle of cercus. Spermatheca small, rounded.

#### Elevation range.

From the sea level to nearly 1700 m.

#### Period of activity.

From the end of April through late July.

#### Habitat.

Mountainous river margins covered with deciduous trees and shrubs.

#### General distribution.

Species was known only from Honshu island, Japan. Recorded from the Korean Peninsula for the first time.

### ﻿ Adelphomyia macrotrichiata

Taxon classificationAnimaliaDipteraLimoniidae

(Alexander, 1923)

A34FE3C3-5F05-5B2A-8CE8-0B13B1BFD860

Figs 22 , 23

Limnophila (Lasiomastix) macrotrichiata Alexander, 1923: 65–66.
Limnophila (Adelphomyia) macrotrichiata : Alexander 1940a: 49, 75, pl. 1, fig. 7.
Adelphomyia macrotrichiata : Savchenko 1983: 52–53; Oosterbroek 2024.

#### Type material examined.

**Japan • Holotype** ♂; wing and genitalia slide-mounted; Hokkaido, Teshio; 3 July 1916; T. Issiki leg.; USNM.

#### Other examined material.

(all these specimens are *A.punctum* but misidentified as *A.macrotrichiata*). **North Korea** • 1 ♂ (wing and genitalia slide-mounted); Ompo; alt. 37 m; 15 June 1937; A. M. Yankovsky leg.; C. P. Alexander det.; USNM • 1 ♀ (pinned); Ompo; alt. 61 m; 20 May 1938; A. M. Yankovsky leg.; C. P. Alexander det.; USNM • 1 ♀ (pinned); Ompo; alt. 61 m; 24 May 1938; A. M. Yankovsky leg.; C. P. Alexander det.; USNM • 1 ex. (pinned, wing and tip of abdomen missing); Ompo; alt. 61 m; 28 May 1938; A. M. Yankovsky leg.; C. P. Alexander det.; USNM • 1 ♂ (pinned); Ompo; alt. 152 m; 28 May 1938; A. M. Yankovsky leg.; C. P. Alexander det.; USNM • 1 ♂ (pinned); Ompo; alt. 91 m; 29 May 1938; A. M. Yankovsky leg.; C. P. Alexander det.; USNM • 2 ♀ (pinned); Ompo; alt. 122 m; 29 May 1938; A. M. Yankovsky leg.; C. P. Alexander det.; USNM.

#### Redescription.

Body semi-polished brownish yellow with darker abdomen. Male body length 5.5–6.8 mm, female 7.7–9.2 mm, male wing length 6.2–9.1 mm, female 7.6–8.5 mm.

***Head.*** Pale bluish grey because of dense pruinosity, covered with long, semi-erect, brownish yellow setae. Eyes widely separated in both sexes, distance between eyes at base of antenna exceeds length of scape. Antenna rather long, approximately reaching to base of halter if bent backwards. Male antenna 1.4 mm long, that of female 1.1–1.6 mm. Scape elongate, nearly cylindrical, obscure yellow, turning brownish towards apex, covered with sparse greyish pruinosity. Pedicel obscure yellow to brown, depending on specimen, wider distally. Few basal flagellomeres yellow to greyish yellow, distal flagellomeres greyish brown. Basal flagellomeres oval, distal segment spindle-shaped. Rostrum brownish, sparsely dusted with grey dorsally, palpus brown.

***Thorax.*** Pronotum pale grey, covered with short erect yellow setae, postpronotum obscure yellow. Presutural scutum semi-polished, uniformly brownish yellow medially, yellowish along frontal and lateral margins, without stripes. Scutal lobe brownish yellow with paler margins. Area between scutal lobes yellow. Scutellum greyish yellow. Mediotergite greyish with yellowish lateral and posterior margins. Pleuron pale brown, sparsely covered with bluish grey pruinosity. Wing (Fig. 22) semi-translucent, yellowish. Stigma oval, pale brown. Indistinct darker areas surround cord, distal margin of discal cell and *m-cu*. Veins pale brown, yellowish at wing base. Venation: *Sc* comparatively long, reaching costal vein slightly before branching point of *Rs*, *sc-r* ~ 3× its own length from tip of *Sc. Rs* long, slightly arched at base. Free end of *R_1_* longitudinal, *R_2_* oblique, 2.8× its own length from tip of *R_1_*. *R_3_* and *R_4_* slightly arched and diverging towards wing margin, cell *r_3_* with short stem, veins *R_4_* and *R_5_* converging towards wing margin. Cross-vein *r-m* distinct, at base of discal cell. Discal cell 2.2× as long as wide. Cell *m_1_* 2.2× as long as its stem. Cross-vein *m-cu* slightly before middle of discal cell. Anal vein distinctly arched at wing margin, ending beyond base of *Rs.* Anal angle widely rounded. Distal radial and medial wing cells with abundant macrotrichiae, covering mostly distal half of each cell, and missing or nearly missing on basal half. Length of male halter 1.0 mm, of female 0.9–1.2 mm. Halter pale, base, and knob slightly infuscate. Coxae obscure yellow, dusted with grey, fore coxa brownish at base. Trochanters pale yellow. Femur pale yellow with slightly darkened brownish apex, tibia yellow with slightly infuscate tip, first tarsomere yellowish brown at base, brown at distal half, remaining tarsomeres brown to dark brown. Male femur I: 4.1–4.4 mm long, II: 5.2 mm, III: 4.3 mm, tibia I: 4.4–5.3 mm, II: 3.8 mm, III: 4.2 mm, tarsus I: 4.5–5.4 mm, II: 4.4 mm, III: 3.9 mm. Female femur I: 4.0–4.5 mm long, II: 4.4 mm, III: 4.6 mm, tibia I: 4.4–4.5 mm, II: 3.7 mm, III: 5.0 mm, tarsus I: 4.0–4.7 mm, II: 4.1 mm, III: 4.2 mm. Claw simple, black, without spines.

***Abdomen.*** Tergites brown, pregenital tergite dark brown. Four basal sternites yellow to pale yellow, remaining sternites brown, pregenital sternite darker. Male terminalia (Fig. 23) with base of ninth segment darker brown. Distal margin of ninth segment, gonocoxites and gonostyli yellow except blackened distal part of outer gonostylus. Epandrium with two apically blunt lobes at the middle of posterior margin and narrow U-shaped indentation between them. Gonocoxite distinctly wider at base, narrower beyond middle, without additional lobe. Outer gonostylus with long, narrow, slightly curved outer branch, and subbasal widening; widened part rounded, but not extended into separate lobe. Outer branch with sclerotised distal part and blackened apex, two small, apical, hook-shaped teeth at tip of outer margin. Inner gonostylus two-branched, outer branch long, narrow, blunt apex knob-shaped, inner branch short, narrow, reaching to approximately middle of outer branch. Aedeagus long, narrow, strongly curved ventrally, distal part at ~ 90° angle to basal part (clearly visible in lateral view). Paramere posteriorly narrow, rod-shaped. Ovipositor obscure yellow. Cercus very long, narrow, distal part slightly raised upwards. Hypogynial valve long, spine-shaped, apex reaching distinctly beyond middle of cercus.

#### Elevation range.

Unknown.

#### Period of activity.

Adults were collected only during first two weeks of July in Japan and the Far East of Russia.

#### Habitat.

Adults are flying among dense grassy vegetation along margins of streams and rivers surrounded by wet broad-leaved forests in South Primorye close to the border with Korea (Savchenko 1983).

#### General distribution.

Species was described from Japan (Hokkaido Island), it is recorded from the Far East of Russia (Primorsky Kray).

#### Remarks.

Wing illustrated in Alexander (1940a: pl. 1, fig. 7) does not belongs to the genus *Adelphomyia* or even to the subfamily Limnophilinae. *Adelphomyiamacrotrichiata* wing venation is probably shown in pl. 1 fig. 15. Savchenko (1983: 52) wrote that macrotrichiae nearly completely cover distal radial and medial cells in the specimens from the Far East of Russia (in ‘Key for identification of regional species’), while North Korean specimens have macrotrichiae mostly at distal half of each cell, basal half bare. Because of that character, North Korean specimens could be identified as *A.punctum* in Savchenko’s key for the Far Eastern *Adelphomyia*. Specimens from the Far East have trichiation more similar to the specimens from Japan and to holotype. Savchenko also mentions a large variability of wing venation, especially in the position of *R_2_*, comparative length of cell *m_1_* and position of *m-cu*. Shape of *A.macrotrichiata* aedeagus is very different from that of *A.punctum*. Aedeagus of *A.macrotrichiata* is long and strongly curved with distal part at right angle to the basal part when aedeagus of *A.punctum* is short and nearly straight. Genitalia of all specimens on which was based the record of *A.macrotrichiata* from North Korea were examined and all of them were identified as *A.punctum*. No *A.macrotrichiata* was found in additional material from the same locality, and the species was not found among other *Adelphomyia* specimens from Korea. Based on this we delete *A.macrotrichiata* from Korean species list. On the other hand, *A.macrotrichiata* was found in the Far East of Russia close to the border with Korea and we expect this species at least in the northern part of the Peninsula.

### ﻿ Adelphomyia punctum

Taxon classificationAnimaliaDipteraLimoniidae

(Meigen, 1818)

E3EA8902-F498-548D-A198-A1683865E7CE

Figs 29–33

Limnobia punctum Meigen, 1818: 128.
Limnophila punctum : Verrall 1886: 200; Meijere 1921: 65, 81; Pierre 1924: 121, 126; Nielsen 1925: 75; Lackschewitz 1940: 85.
Adelphomyia helvetica : Bergroth 1891: 134–135; Pierre, 1924: 115.
Limnophila (Adelphomyia) punctum : Alexander 1938: 324.
Adelphomyia punctum : Savchenko 1986: 275; Savchenko 1989: 79.

#### Examined material.

(Fig. 49) **North Korea** • 2 ♂ (pinned); Ompo; alt. 76 m; 19 May 1937; Yankovsky leg.; USNM • 1 ♀ (pinned); Ompo; alt. 76 m; 9 June 1937; Yankovsky leg.; USNM • 1 ♂, 8 ♀ (pinned); Ompo; alt. 37 m; 15 June 1937; Yankovsky leg.; USNM • 1 ♂, 1 ex. (broken, pinned); Ompo; alt. 183 m; 23 June 1937; Yankovsky leg.; USNM • 1 ♂ (pinned); Ompo; alt. 274 m; 12 May 1938; Yankovsky leg.; USNM • 1 ♂ (pinned); Ompo; alt. 305 m; 13 May 1938; Yankovsky leg.; USNM • 1 ♂ (pinned); Ompo; alt. 46 m; 25 May 1938; A. Yankovsky leg.; USNM • 1 ♂ (pinned); Ompo; alt. 122 m; 27 May 1938; Yankovsky leg.; USNM • 1 ♂, 1 ♀ (pinned); Ompo; alt. 91 m; 29 May 1938; Yankovsky leg.; USNM • 1 ♂, 5 ♀ (pinned); Ompo; alt. 122 m; 3 June 1938; Yankovsky leg.; USNM • 1 ♀ (pinned); Ompo; alt. 91 m; 9 June 1938; Yankovsky leg.; USNM • 1 ♂, 3 ♀ (pinned); Ompo; alt. 91 m; 10 June 1938; Yankovsky leg.; USNM • 3 ♀ (pinned); Ompo; alt. 107 m; 11 June 1938; Yankovsky leg.; USNM • 3 ♂, 2 ♀ (pinned); Seren Mts.; alt. 762 m; 14 June 1938; A. Yankovsky leg.; USNM • 2 ♂ (pinned); Seren Mts.; alt. 853 m; 15 June 1938; Yankovsky leg.; USNM • 1 ♂ (pinned); Seren Mts.; alt. 914 m; 22 June 1938; A. Yankovsky leg.; USNM • 2 ♂, 1 ex. (broken, pinned); Seren Mts.; alt. 1067 m; 22 June 1938; A. Yankovsky leg.; USNM • 1 ♂ (pinned); Seren Mts.; alt. 1219 m; 22 June 1938; A. M. Yankovsky leg.; USNM • 1 ♀ (pinned); Seren Mts.; alt. 1524–1829 m; 25 June 1938; A. M. Yankovsky leg.; USNM • 1 ♀ (pinned); Seren Mts.; alt. 1676 m; 25 June 1938; A. Yankovsky leg.; USNM • 2 ♂, 1 ♀ (pinned); Seren Mts.; alt. 1829 m; 25 June 1938; A. Yankovsky leg.; USNM • 1 ♂ (pinned); Seren Mts.; alt. 914 m; 30 June 1938; A. Yankovsky leg.; USNM • 1 ♂ abdomen (pinned); Kankyo Nando Puksu Pyaksan; alt. 1829 m; 15 June 1939; Yankovsky leg.; USNM • 1 ♂ (pinned, genitalia dissected in microvial with glycerol on same pin); Kankyo Nando, Puksu Pyaksan; alt. 1676 m; 17 July 1939; A. Yankovsky leg.; USNM • 1 ♂, 1 ♀ (pinned); Kankyo Nando, Puksu Pyaksan; alt. 1829 m; 24 July 1939; A. Yankovsky leg.; USNM • 1 ♂, 2 ♀ (pinned); Kankyo Nando, Puksu Pyaksan; alt. 1524 m; 3 August 1939; A. Yankovsky leg.; USNM; **South Korea** • 1 ♂ (in ethanol); Gangwon-do, Pyeonchang-gun, Odaesan National Park; 37.74913°N, 128.57723°E; alt. 726 m; 22 June 2012; S. Kim, S. Podenas leg.; NIBR • 2 ♂ (in ethanol); Jeollabuk-do, Namwon, Unbong-eup, Hwasu-ri; 35.45345°N, 127.57759°E; alt. 509 m; 6 May 2013 (01); S. Podenas leg.; NIBR • 1 ♂ (in ethanol); Gyeongsangnam-do, Hamyang, Macheon-myeon, Samjeong-ri; 35.36713°N, 127.65228°E; alt. 406 m; 11 May 2013 (5); S. Podenas leg.; NIBR • 2 ♂ (in ethanol); Gyeonggi-do, Paju-si, Gunnae-myeon, Jeomwon-ri, Gate (South-MDL); 37.93430°N, 126.72097°E; alt. 39 m; 20 May 2016; T. E. Klein, H.-C. Kim leg.; Mosquito Magnet; NIBR • 1 ♀ (in ethanol); Jeollanam-do, Gurye-gun, Toji-myeon, Naeseo-ri, Piagol valley; 35.27333°N, 127.56924°E; alt. 546 m; 3 June 2016 (3); S. Podenas leg.; NIBR • 1 ♀ (in ethanol); Jeollanam-do, Gurye-gun, Toji-myeon, Naeseo-ri, Piagol valley; 35.27123°N, 127.57133°E; alt. 534 m; 4 June 2016 (1); V. Podeniene leg.; NIBR • 2 ♂ (in ethanol); Gyeonggi-do, Paju-si, Gunnae-myeon, Jeomwon-ri, Gate (South-MDL); 37.93431°N, 126.72096°E; alt. 39 m; 17 May 2019; T. E. Klein, H.-C. Kim leg.; Mosquito Magnet; NIBR.

#### Comparative material examined.

**Lithuania** • 1 ♂ (genitalia in microvial with glycerol); Raseiniai district, Sargeliai; 55.4762°N, 23.4563°E; 5–13 June 2009; NRC • 1 ♂, 1 ♀ (pinned); Moletai distr., river Skardis; 55.29132°N, 025.45485°E; alt. 150 m; 26 May 2012; S. Podenas leg.; NRC.

#### Redescription.

General body colouration varies from yellowish brown to greyish brown. Body length of male 3.9–8.2 mm, female 6.4–7.2 mm. Male wing: 6.1–8.8 mm; female wing: 6.1–8.3 mm.

***Head.*** Slightly extended posteriorly, grey, brownish grey postero-laterally, pale grey frontally, covered with long, sparse, yellowish setae, longest of which nearly as long as both basal antennomeres combined. Eyes widely separated in both sexes, distance between them at base of antennae nearly same as length of both basal antennomeres together. Length of male antenna 0.9–1.8 mm, reaching wing base if bent backward; female 1.1–1.3 mm. Scape brown dusted with grey, elongate, nearly cylindrical, 2.2× as long as wide and 2× as long as pedicel. Pedicel oval, brown, covered with few short setae. Flagellum 14-segmented, brown, distal flagellomeres darker. Flagellomeres oval with short apical pedicels, apical segment nearly as long as preceding. Verticils up to 2.5× as long as respective segments. Short erect pubescence, covering segments pale. Rostrum, palpi, and mouth parts dark brown to blackish.

***Thorax.*** Cervical sclerites brown, dark brown ventrally, covered with grey pruinosity. Thorax yellowish brown to brown covered with sparse grey pruinosity. Pronotum pale brown to brown, dusted with grey, dorso-laterally covered with sparse erect long setae. Mesonotal prescutum semi-polished, pale brown, sparsely dusted with grey, yellowish laterally, covered with sparse erect setae, longitudinal stripes absent or very indistinct in some specimens. Tubercular pit small, brown, close to frontal margin of sclerite, pseudosutural fovea distinct dark brown. Scutal lobe frontally concolourous with presutural scutum, laterally and posteriorly brownish yellow. Area between scutal lobes greyish. Scutellum brownish at the middle, yellow laterally. Mediotergite yellow with narrow indistinct greyish brown median line. Pleuron bare, without setae, brownish yellow, sparsely dusted with grey; posterior basal area brown. Wing (Fig. 29) semi-translucent, with indistinct darkening surrounding cross-veins and apices of all longitudinal veins at wing margin, some specimens without such darkening. Stigma elongate, brownish. Veins brown, yellowish at wing base and in costal area. Macrotrichiae more abundant in radial cells and cell *m_1_*, present also in other marginal cells along postero-apical wing margin. Venation: *Sc* long, but not reaching branching point of *Rs*, *sc-r* ~ 3× its own length from tip of *Sc. Rs* long, slightly arched or angulate at base, with or without short spur. Free end of *R_1_* longitudinal, *R_2_* transverse, indistinct, ~ 2× its own length from tip of *R_1_*, *R_3_*, and *R_4_* slightly diverging towards wing margin, cell *r_3_* with short stem. Cross-vein *r-m* distinct, at base of discal cell. Discal cell 2× as long as wide. Cross-vein *m-cu* slightly before middle of discal cell. Anal vein slightly arched at wing margin, ending slightly before base of *Rs.* Anal angle widely rounded. Male halter 0.9–1.7 mm long, female 0.8–1.3 mm. Halter pale brownish, knob slightly infuscate distally. Frontal coxa pale brown frontally, yellow posteriorly, remaining coxae yellow, slightly dusted with grey, covered with long erect yellowish setae. Trochanters yellow to brownish yellow. Femur yellow, darkened at apex, tibia yellowish brown with darkened distal part, tarsomeres dark brown, only base of basal tarsomeres yellowish. Male femur I: 3.9–5.0 mm, II: 4.2–5.2 mm, III: 4.2–6.2 mm, tibia I: 5.7–5.9 mm, II: 4.8–5.5 mm, III: 4.5–6.2 mm, tarsus I: 5.7–6.4 mm, II: 5.4–5.9 mm, III: 3.9–5.3 mm. Female femur I: 3.6 mm long, II: 3.8–4.2 mm, III: 4.1–4.7 mm, tibia I: 4.1 mm, II: 3.5–4.0 mm, III: 4.0–5.0 mm, tarsus I: 3.9–4.0 mm, II: 3.5–3.8 mm, III: 3.1–3.7 mm long. Claw simple without subbasal spines or teeth.

***Abdomen.*** Semi-polished brownish yellow to brown. Two pregenital segments darkened in male, concolourous with the rest abdominal segments in female. Tergites with narrowly darkened lateral margins, with two transverse narrow indentations frontally and slightly paler posterior margin in both sexes. Male terminalia (Figs 30, 31) brownish yellow. Ninth tergite with two triangle-shaped lobes at the middle of posterior margin. Gonocoxite elongate, distinctly wider at base, narrower beyond middle, without additional lobe. Outer gonostylus with long narrow outer branch and small rounded lobe at base. Outer branch with sclerotised distal part and blackened apex, two small apical hook-shaped teeth curved medially. Inner gonostylus large, fleshy, setose, two-branched. Outer branch long and narrow, inner branch narrowly triangle-shaped reaching approximately to middle of outer branch. Aedeagus (Fig. 32) long and nearly straight, paramere posteriorly short, slightly arched, reaching before middle of aedeagus. Ovipositor (Fig. 33) yellow with very long, narrow cercus and hypovalva. Distal part of cercus slightly turned upwards, dorsal margin brownish, apex point-shaped. Hypogynial valve long, straight, wide at base, narrowing towards apex, distal part distinctly narrower, apex reaching beyond middle of cercus, dorsal margin at ~ 1/4 length from apex with long dense setae.

#### Elevation range.

From the lowest elevations in Korea to more than 1800 m.

#### Period of activity.

Adults on wing from beginning of May through early August.

#### Habitat.

Wet places near streams and ponds surrounded by deciduous trees and in wet places of deciduous and mixed forests.

#### General distribution.

Species has widely disjunct area, it is widely distributed in Europe, recorded also from eastern part of East Palaearctic. Recorded from the Korean Peninsula for the first time.

#### Remarks.

Some specimens from North Korea are distinctly darker than specimens from Europe, their wings lack any pattern except stigma. At the moment, it is difficult to say if that is variation or colour change due to long preservation. North Korean specimens were collected 80 years ago. We examined male genitalia of these specimens and compared them with “typical” specimens from Europe and South Korea, but no differences were observed.

### ﻿ Adelphomyia jejuana

Taxon classificationAnimaliaDipteraLimoniidae

Podenas sp. nov.

215C5FF6-8FA9-56AC-BDE6-30E8F162A58E

https://zoobank.org/5EA7A898-2697-49FE-B459-C68920F6C199

Figs 40–43 , 44–46

#### Type material examined.

**South Korea • Holotype** ♂ (pinned, wing slide-mounted, genitalia in microvial with glycerol on same pin); Jeju-do, Jeju-si, Hallasan National Forest; 33.43222°N, 126.59776°E; alt. 577 m; 24 May 2017 (1); S. Podenas leg.; NIBR. **Paratypes** • 1 ♀ (pinned, wing slide-mounted); same data as holotype, topotypic; NIBR • 1 ♀ (in ethanol); Jeju-do, Seogwipo-si, Saekdal-dong; 33.36044°N, 126.46275°E; alt. 1103 m; 19 June 2019 (1); S. Podenas leg.; NIBR.

#### Diagnosis.

Reddish brown species ~ 4–7 mm long (Figs 40, 44). Head greyish brown, thorax pale brown, prescutal stripes missing. Wing brownish, no pattern with indistinct stigma. Abdomen yellow, dorsally slightly darker than ventrally. Ninth tergite of male genitalia with two point-apexed triangle-shaped lobes at posterior margin. Gonocoxite elongate. Outer gonostylus with long narrow outer branch and small angulate lobe at base. Outer branch with longitudinal wrinkles, sclerotised distal part and blackened apex. Distal part with widely rounded medial edge and two small apical hook-shaped teeth curved medially. Inner gonostylus large, fleshy, setose, two-branched. Aedeagus comparatively short, slightly arched, paramere narrowly rod-shaped, slightly arched, reaching to ~ 2/3 of aedeagus length. Ovipositor yellow with very long, narrow cercus and hypovalva. Apical part of cercus slightly turned upwards.

#### Etymology.

Species is named after its type locality, Jeju Island, Korea.

#### Description.

General body colour reddish brown (Figs 40, 44). Body length of male ~ 4 mm, female 5.3–6.8 mm. Male wing: 6.2 mm, female wing: 6.7 mm.

***Head.*** Greyish brown, posteriorly pale brown, pale grey along eye margin, with few yellowish setae dorsally. Eyes widely separated in both sexes, distance between them at base of antennae same as length of scape. Length of female antenna 1.2 mm, reaching wing base if bent backwards. Scape brown dusted with grey, elongate, nearly cylindrical, 2× as long as wide, and 2× as long as pedicel. Pedicel rounded, brown, covered with few short setae. Flagellum 14-segmented, pale brown. Flagellomeres oval with short apical pedicels, apical segment as long as preceding. Verticils 2× as long as respective segments. Short erect pubescence, covering segments pale. Rostrum, palpi, and mouth parts brown sparsely dusted with grey.

***Thorax.*** Brownish yellow, covered with sparse brownish grey pruinosity. Cervical sclerites and pronotum brown sparsely dusted with grey. Pronotum elongate with extended postero-lateral angle. Mesonotal prescutum semi-polished, brownish yellow with sparse grey pruinosity, frontal margin slightly darkened, stripes missing. Scutal lobes, scutellum, and mediotergite uniformly brownish yellow. Pleuron brownish yellow indistinctly darker above coxae. Wing (Figs 41, 45) translucent with weak brownish tint, slightly yellowish at base. No darkening along cross-veins or branching points of veins. Stigma indistinct, nearly missing. Veins pale brown. Wing venation: vein *Sc* long, apex reaching wing margin slightly before branching point of radial sector, *sc-r* 2× its own length before apex of *Sc*, *Rs* long, nearly straight, just slightly arched at base, *R_2_* 4× its own length before apex of *R_1_*, cell *r_1_* slightly widened at wing margin; *R_3_* and *R_4_* slightly diverging towards wing margin, cell *r_3_* with short stem. Cross-vein *r-m* distinct, at base of discal cell. Discal cell 2.3× as long as wide. Cross-vein *m-cu* slightly before middle of discal cell. Anal vein slightly arched at wing margin, ending at the level of *Rs* base in male, slightly beyond base of *Rs* in female. Anal angle widely rounded. Male halter 0.8 mm long, female 1.0 mm. Halter brownish yellow, knob with greyish tinge. Coxae and trochanters brownish yellow, legs yellow with brownish distal tarsomeres. Male femur I: 3.6 mm, III: 4.5 mm, tibia I: 4.7 mm, III: 4.6 mm, tarsus I: 4.7 mm, III: 4.0 mm. Female femur I: 3.7 mm long, II: 3.5 mm, III: 4.2 mm, tibia I: 4.0 mm, II: 3.5 mm, III: 4.2 mm, tarsus I: 4.0 mm, II: 3.5 mm, III: 3.5 mm long. Claw simple without subbasal spines or teeth.

***Abdomen.*** Tergites brownish yellow, sternites yellow. Male terminalia (Fig. 42) yellow. Ninth tergite with two sharply apexed triangle-shaped lobes at the middle of posterior margin and wide V-shaped emargination between them. Gonocoxite elongate, wider at base, narrower beyond two thirds of length, without additional lobe. Outer gonostylus with long, narrow outer branch and small angulate lobe at base. Outer branch with longitudinal wrinkles, sclerotised distal part and blackened apex. Distal part with widely rounded medial edge and two small apical hook-shaped teeth curved medially. Inner gonostylus large, fleshy, setose, two-branched. Outer branch long, narrow, inner branch short triangle-shaped reaching to ~ 1/4 of outer branch length. Aedeagus (Fig. 43) comparatively short, slightly arched, paramere narrowly rod-shaped, slightly arched, reaching to ~ 2/3 of aedeagus. Distal part of interbase pale yellow, rounded. Ovipositor (Fig. 46) yellow with very long narrow cercus and hypovalva. Apical part of cercus slightly raised upwards. Hypogynial valve wide at base, apical part distinctly narrower, dorso-apical margin covered with long setae. Apex of hypogynial valve reaches slightly beyond middle of cercus.

#### Distribution.

Currently known only from Jeju Island, South Korea (Fig. 50).

#### Habitats.

Small valley of temporary stream covered with deciduous trees and shrubs, moss-covered rocks; small swampy meadow on the edge of small stream surrounded by deciduous forest.

#### Elevation.

From less than 600 m to 1100 m.

#### Period of activity.

Adults on wing from late May through middle of June.

#### Remarks.

*Adelphomyiajejuana* Podenas, sp. nov., having unpatterned wings and pale body colouration, resembling *A.flavella* but differs from it by details of male terminalia and darker body colouration. Ninth tergite of *A.jejuana* Podenas, sp. nov., especially lobes on posterior margin, resemble that of *A.acicularisbidens*, but in *A.jejuana* Podenas, sp. nov. they are distinctly wider at base and point-apexed. Subapical angle of outer gonostylus is low and widely rounded in *A.jejuana* Podenas, sp. nov., when that in most other Palaearctic species is very distinct and nearly right-angled. Aedeagus of *A.jejuana* Podenas, sp. nov. is shorter than that in *A.flavella*, just slightly extending beyond apices of parameres, when that in *A.flavella* is very long and distinctly curved. Aedeagus in *A.acicularisbidens*, *A.saitamae*, *A.macrotrichiata*, *A.breviramus*, *A.biacus*, and *A.flavella* is strongly curved at nearly right angle, straight in *A.punctum*, *A.casiella*, and *A.satsumicola*, but slightly arched in *A.jejuana* Podenas, sp. nov. Paramere of *A.jejuana* Podenas, sp. nov. is similar to that of *A.flavella*.

##### ﻿Other examined material from Palaearctic

*Adelphomyiaacicularisacicularis* (Alexander, 1954) (Figs 1–3). **Japan • Holotype** ♂; as Limnophilla (Adelphomyia) acicularis; wing, leg, and genitalia slide-mounted; Shikoku, Mt. Tsurugi-Awa; 1 June 1950; Issiki-Ito leg.; USNM; • **Paratype** ♂; head, wing, leg, and genitalia slide-mounted; Shikoku, Imanoyama, Tosa; alt. 865 m; 12 May 1951; Issiki-Ito leg.; USNM.

*Adelphomyiabiacus* (Alexander, 1954) (Figs 9–11). **Japan** as Limnophila (Adelphomyia) biacus; • **Holotype** ♂; slide–mounted; Shikoku, Mt. Isizuti; June 10, 1950; Issiki-Ito leg.; USNM; • **Paratype** ♂; slide-mounted; Shikoku, Omogokei; June 6, 1952; T. Yano leg.; USNM.

*Adelphomyiabreviramus* (Alexander, 1924) (Figs 12–14). **Japan** as Limnophila (Lasiomastix) breviramus; • **Holotype** ♂; slide-mounted; Yumoto; alt. 1774 m; 23 July 1923; T. Esaki leg.; USNM; • **Metatype** ♂; slide-mounted; Shikoku, Mt. Ishizuchi-Iyo; 9 June 1950; Issiki Ito leg.; USNM; as Limnophila (Adelphomyia) brevirama; • **Metatype** ♂; slide-mounted; Hida, Ontake; 26 July 1959; T. Mishima leg.; USNM.

*Adelphomyiacaesiella* (Alexander, 1929) (Figs 15, 16). **Japan** as Limnophila (Tricholimnophila) caesiella; • **Metatype** ♂; slide-mounted; Kiushiu, Mt. Kirishima; alt. 762 m; 3 May 1929; S. Issiki leg.; USNM.

*Adelphomyiapilifer* (Alexander, 1919) (Figs 24–26). **Japan • Paratopotype** ♂; as Limnophila (Lasiomastix) pilifer; slide-mounted wing and genitalia; Tokyo, Meguro; 9 April 1919; R. Takahashi leg.; USNM • 1 ♂; pinned; Hokkaido, near Sapporo, Maruyama; 31 May 1953; S. Kuwayama leg.; USNM • 2 ♂, 1 specimen with broken abdomen; pinned; Hokkaido, Prov. Ishikari, Nopporo; 18 June 1953; Y. Nishio leg.; USNM.

*Adelphomyiaprionolaboides* (Alexander, 1934) (Figs 27, 28). **Japan** as Limnophila (Adelphomyia) prionolaboides; • **Metatype** ♂; slide-mounted; Mino, Sakauchi; 4 May 1958; T. Mishima leg.; USNM.

*Adelphomyiasaitamae* (Alexander, 1920) (Figs 34, 35). **Japan** as *Limnophilasaitamae*; • **Metatype** ♂; slide-mounted; Honshu, Tyuzenzi; 22 June 1932; S. Issiki leg.; USNM.

*Adelphomyiasatsumicola* (Alexander, 1930) (Figs 36, 37). **Japan** as Limnophila (Tricholimnophila) satsumicola; • **Holotype** ♂; slide-mounted; Shiroyama hill, city-oz Kagoshima; 27 April 1929; S. Issiki leg.; USNM; • **Paratype** ♂; slide-mounted, Shiroyama hill, Kagoshima city; 27 April 1929; S. Issiki leg.; USNM.

*Adelphomyiasimplicistyla* (Alexander, 1940b) (Figs 38, 39). **China** as *Limnophilasimplicistyla*; • **Holotype** ♂; slide-mounted; Szechwan [Sichuan], Omei, Nwa Ien Ting Temple; alt. 1981 m; 15 June 1938; Tsen leg.; USNM.

## Supplementary Material

XML Treatment for Adelphomyia

XML Treatment for Adelphomyia acicularis bidens

XML Treatment for Adelphomyia flavella

XML Treatment for Adelphomyia macrotrichiata

XML Treatment for Adelphomyia punctum

XML Treatment for Adelphomyia jejuana

## ﻿Additional information

### Author ORCIDs

Sun-Jae Park https://orcid.org/0000-0003-3759-6517

Sigitas Podenas https://orcid.org/0000-0002-2597-566X

### Data availability

All of the data that support the findings of this study are available in the main text.
